# Habitat Features Influencing Waterbird Use of Managed Wetlands Enrolled in a Public‐Private Partnership for Land Conservation: The California Waterfowl Habitat Program

**DOI:** 10.1002/ece3.72032

**Published:** 2025-09-18

**Authors:** C. Alex Hartman, Joshua T. Ackerman, Sarah H. Peterson, Brady L. Fettig, Mark P. Herzog

**Affiliations:** ^1^ U.S. Geological Survey Western Ecological Research Center, Dixon Field Station Dixon California USA

**Keywords:** density, emergent vegetation, land cover, shorebirds, water depth, waterfowl

## Abstract

Draining, water diversion, and development have greatly reduced the availability of freshwater wetland habitat around the world, and many remaining wetlands are on private lands. Public–private partnership programs can be an important means for promoting habitat conservation and management on private lands. We investigated bird use of 117 wetlands enrolled in the California Waterfowl Habitat Program in California's Central Valley, where two‐thirds of wetlands are under private ownership and management. Specifically, we quantified the influence of wetland habitat features and surrounding land cover on waterbird density and diversity in late winter and early spring and during the waterfowl breeding season. Dabbling duck and shorebird densities were highest in wetlands that had water depths < 20 cm, and waterbird densities decreased with water depth. Greater amounts of emergent vegetation, especially tall and dense emergent vegetation, had a negative effect on total waterbird density but a positive effect on species richness and secretive marsh bird density. Shorebird and breeding duck densities were lower in wetlands with a large number of trees and other potential perch sites, and waterbird densities decreased with the amount of nearby wetland habitat on the landscape. Overall, we estimated that during late winter and early spring, private properties that were enrolled in the California Waterfowl Habitat Program (8000–8500 ha each year) supported 480,000 birds per day during extreme drought conditions in 2022 and 280,000 birds per day in more normal, non‐drought conditions in 2023. Over the 76‐day winter and early spring survey period, this amounted to more than 20 million bird use days on wetlands enrolled in the California Waterfowl Habitat Program during late winter and early spring. These results demonstrate the value of public–private wetland conservation partnerships, the influence of wetland habitat features and surrounding land cover on waterbird abundance, and the benefits of habitat features that could be incorporated into management plans and wetland selection criteria for enrollment into public–private conservation programs.

## Graphical abstract

Public–private partnership programs can be an important means for promoting wetland conservation and management on private lands. We investigated bird use of 117 wetlands enrolled in the California Waterfowl Habitat Program in California's Central Valley. Results demonstrate the value of public–private wetland conservation partnerships, the influence of wetland habitat features and surrounding land cover on waterbird abundance, and how habitat features could be incorporated into management plans and wetland selection criteria for enrollment into public–private conservation programs.

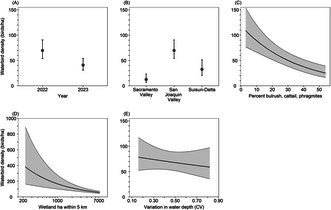

## 1 Introduction

Wetlands are important and productive ecosystems that provide critical services, including maintaining water quality, flood protection, and carbon sequestration (Mitsch and Gosselink 2015). Wetlands also support high levels of biodiversity. Although they comprise only 1% of the earth's surface area, freshwater wetlands account for more than 40% of the world's species (Hu et al. 2017). Yet, widespread draining, water diversion, and development have resulted in worldwide wetland losses of as much as 87% (Davidson 2014), including more than 50% of wetland habitats in the contiguous United States (Dahl and Johnson 1991). Moreover, increased frequency of water shortages and droughts due to climate change and greater demand on freshwater sources by growing populations complicate efforts to protect wetland habitats (Schewe et al. 2014).

The large and continuing loss of wetland habitat is a major conservation concern for many wildlife species. Approximately one‐half of the threatened and endangered species in the United States are wetland dependent (Lang et al. 2024). In addition, waterbirds, particularly waterfowl and shorebirds, are highly dependent on wetland habitats for breeding, wintering, and migration. Protecting and managing wetlands on public lands, including National Wildlife Refuges and State Wildlife Areas, is an important component of maintaining wetland habitat for waterbirds and other wildlife. Yet, approximately 75% of the wetlands in the contiguous United States are privately owned (U.S. Environmental Protection Agency 2024). Therefore, engagement and collaboration with private landowners are critical for meeting conservation objectives for wetland habitats to support wetland‐dependent species.

Public–private partnerships are an important means for promoting wetland conservation and management on private lands and the species that depend on them (Benson et al. 2019; Brasher et al. 2019). In these partnerships, public entities, such as state or federal agencies, provide private landowners with technical assistance and financial incentives for wetland restoration and management. For example, wetlands could be placed under a conservation easement, limiting some uses of the land that would compromise its conservation value in exchange for monetary compensation. Alternatively, landowners could receive financial incentives in exchange for managing their wetlands in accordance with a plan developed cooperatively with federal or state agency staff. Such public–private partnerships have been shown to be effective at reducing costs associated with private wetland management and have resulted in the restoration or preservation of 7 million ha of wetlands in North America (Benson et al. 2019).

In the arid western United States, the greatest wetland loss has occurred in California, with an estimated 4.5 million fewer acres, a loss of 91% (Dahl 1990). Much of this wetland loss has occurred in California's Central Valley, through water drainage, diking, and diversion in support of agricultural and urban development (Frayer et al. 1989). Because of these losses, the remaining wetlands in California's Central Valley are critically important habitats, especially for many waterbirds. In fact, California's Central Valley annually supports approximately 8.5 million waterfowl during winter (approximately 60% of the Pacific Flyway's waterfowl population) and up to 500,000 shorebirds (Shuford et al. 1998; Ackerman et al. 2014; Central Valley Joint Venture 2020; Skalos and Weaver 2020). Central Valley wetlands that are flooded in the spring and summer also support hundreds of thousands of breeding ducks, primarily mallard ( *Anas platyrhynchos* ), gadwall (*Mareca strepera*), and cinnamon teal (*Spatula cyanoptera*), as well as many breeding shorebirds and other waterbirds (Ackerman et al. 2014; Central Valley Joint Venture 2020). Because the amount of natural wetland habitat has decreased, waterbirds have become increasingly reliant on the protection of restored managed wetlands, as well as certain flooded agricultural crops. In the Central Valley, waterbirds use a wetland habitat mosaic that includes public wetlands (National Wildlife Refuges, State Wildlife Areas), privately managed duck clubs and other private reserves, and compatible agriculture, particularly post‐harvested rice ( *Oryza sativa* ) fields that are flooded during the fall and winter to promote straw decomposition (Fleskes et al. 2005; Ackerman et al. 2006; Central Valley Joint Venture 2020; Donnelly et al. 2022). Private lands account for approximately two‐thirds of wetlands, and public lands account for approximately one‐third of wetlands in the Central Valley (Central Valley Joint Venture 2020).

The California Waterfowl Habitat Program (https://wildlife.ca.gov/Lands/WCP/Private‐Lands‐Programs/Waterfowl‐Habitat) is one example of a public–private partnership that can increase the availability of private wetland habitat for the benefit of private landowners and waterbirds alike (Brasher et al. 2019). Also known as the Presley Program, the California Waterfowl Habitat Program provides economic incentives to private landowners who enroll their seasonal wetlands (flooded in the fall and winter), semi‐permanent wetlands (flooded in the fall through summer), or reverse‐cycle wetlands (flooded in the spring and summer) in the program and follow a wetland management plan designed to improve habitat conditions for wildlife, particularly waterfowl and other waterbirds. Wetland management plans are developed cooperatively between California Department of Fish and Wildlife (CDFW) biologists and the participating landowner and seek to increase food supplies for wintering waterfowl, sustain optimal water depths for foraging by waterfowl and shorebirds, and provide spring and summer‐flooded wetlands near upland nesting habitat for breeding waterfowl and other waterbirds. Wetland management plans include habitat management actions such as irrigation schedules for moist‐soil management to promote growth of desirable food plants and control measures, such as discing, to reduce the amount of unwanted vegetation. For semi‐permanent and reverse‐cycle wetlands, management actions that maintain water for ducklings throughout the brood‐rearing period and enhance upland nesting habitats adjacent to these wetlands are also incorporated.

Identifying management practices that enhance or improve the value of wetland habitats to waterbirds could improve wetland management plans and provide more specific criteria for selecting private wetlands for enrollment in incentive programs. In this study, our objectives were to evaluate the effect of habitat features on waterbird density and diversity in wetlands enrolled in the California Waterfowl Habitat Program, as well as densities of dabbling ducks, shorebirds, wading birds, and secretive marsh birds. These guilds were selected because they are among the most abundant waterbird guilds in Central Valley wetlands, are of particular importance to conservation efforts in the Central Valley (Central Valley Joint Venture 2020), and have varying habitat preferences. Specifically, we examined how bird density and diversity were affected by large‐scale habitat features, including wetland shape, emergent vegetation cover, mudflat cover, and the number of nearby perch sites available to aerial predators. We also examined the influence of small‐scale habitat features, such as water depth, vegetation height, and the variation of these features within wetlands, on bird density and diversity. Finally, we investigated the influence of surrounding land cover types, including the amount and proximity to wetland habitats, agricultural habitats, upland habitats, and developed lands. Surrounding landscape features have been found to influence wildlife use of specific habitat patches (Elphick 2008; Webb et al. 2010; Beatty et al. 2014; Reiter et al. 2015). For example, non‐breeding mallards in the midcontinent of North America selected habitat patches based on proximities to cropland and wetland habitats (Beatty et al. 2014); non‐breeding waterbird densities in rice fields in the Sacramento Valley were influenced by the amount of nearby wildlife refuges, semi‐natural wetland habitat, and flooded rice habitat (Elphick 2008; Reiter et al. 2015). Similarly, proximity to upland nesting habitat may influence breeding waterfowl use of specific wetlands for brood‐rearing habitat because duckling survival is negatively related to the distance that broods must travel between upland nesting habitat and adjacent wetlands (Ball et al. 1975; Peterson, Ackerman, Hartman, et al. 2024). We predicted that wetland habitat attributes that supported the greatest bird densities would vary by guild, with greater dabbling duck and shorebird densities in wetlands with shallower water with little to moderate emergent vegetation and wading bird and secretive marsh bird densities greater in wetlands with more emergent vegetation cover. In addition, we predicted that densities of all waterbird guilds, as well as waterbird species richness, would be greater as the amount of nearby wetland habitat increased, and densities would be lower as the amount of nearby developed and agricultural land increased. Data supporting this manuscript are available as a USGS Data Release (Hartman and Ackerman 2025).

## 2 Materials and Methods

### 2.1 Study Area

**FIGURE 1:**
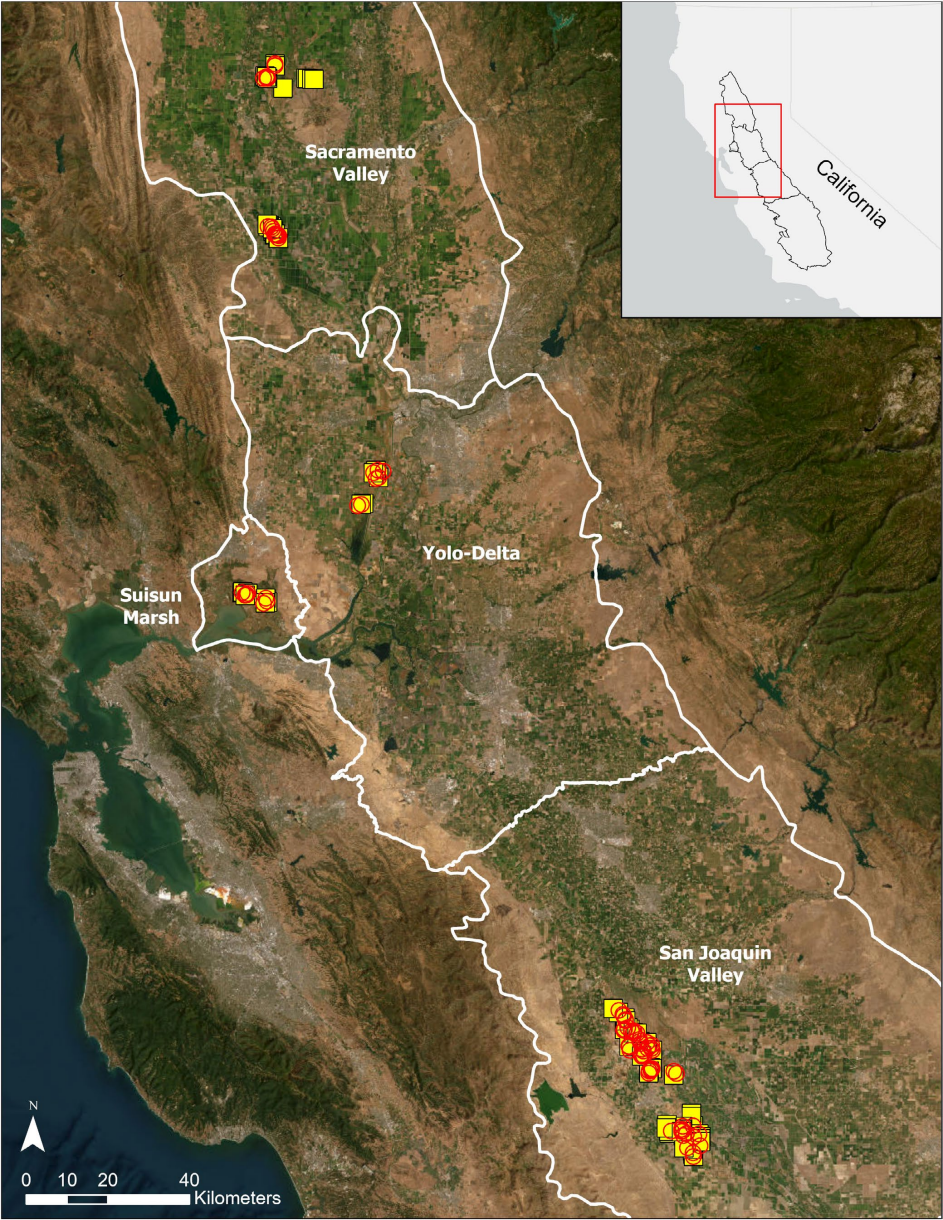
Distribution of wetland units in the Sacramento Valley, San Joaquin Valley, and Suisun‐Yolo‐Delta regions surveyed in Year 1 (2022, yellow squares) and Year 2 (2023, red circles) that were enrolled in the California Waterfowl Habitat Program. Base imagery credits: ArcGIS Map Service (https://services.arcgisonline.com/ArcGIS/rest/services/World_Imagery/MapServer). Region outline credits: Central Valley Joint Venture.

We surveyed flooded wetlands enrolled in the California Waterfowl Habitat Program within three regions: the Sacramento Valley, the San Joaquin Valley, and the Yolo‐Delta and Suisun Marsh area (hereafter Suisun–Delta), from February 2022 through June 2022 (Year 1) and February 2023 through July 2023 (Year 2; Figure 1). In both years, surveys began shortly after the conclusion of the duck hunting season on January 31st. The Sacramento Valley lies at the northern end of the Central Valley and contains several National Wildlife Refuges and State Wildlife Areas, as well as 95% of the rice fields in the Central Valley (Central Valley Joint Venture 2020). The San Joaquin Valley is located approximately in the middle section of the Central Valley, north of the Tulare Basin. The San Joaquin Valley is dominated by agricultural development and contains several National Wildlife Refuges and State Wildlife Areas in addition to many private wetlands in the Grasslands Ecological Area on the west side of the region (Central Valley Joint Venture 2020). Suisun Marsh is a large brackish marsh located in the Sacramento‐San Joaquin River Delta and is an important breeding site for dabbling ducks in California (McLandress et al. 1996; Ackerman et al. 2014; Central Valley Joint Venture 2020). The Yolo‐Delta region is located east of Suisun Marsh and between the Sacramento Valley and San Joaquin Valley. All of these regions experience a Mediterranean climate with warm, dry summers and mild, wet winters. Rainfall is typically higher in the Sacramento Valley than in the San Joaquin Valley.

Permission to access and survey privately owned wetlands was facilitated by the CDFW's Wetland Conservation Program. Logistically, it was not possible to follow an a priori randomized sampling design. Instead, in coordination with CDFW and landowners, we randomly selected and scheduled wetland surveys as wetlands became available (enrolled in the conservation program and flooded). For logistical purposes, we organized surveys into three periods (February–March, April–May, and June–July), and each wetland was surveyed once within a given period and up to three times in a study year (once every survey period). Although some California Waterfowl Habitat Program wetlands are flooded as early as September, we were not permitted to access wetland units to perform surveys until the waterfowl hunting season concluded in late January.

### 2.2 Bird Surveys

Bird surveys consisted of a combination of an area search survey, in which we walked the entire perimeter of the flooded area of the wetland unit, and point count surveys placed at predetermined random locations along the wetland perimeter. Bird surveys were conducted during the morning hours, with most surveys beginning within 30 min after sunrise, and all surveys beginning within 4 h of sunrise. Surveys were not attempted during adverse conditions (e.g., heavy rain or fog, winds > 20mph, on‐site disturbance) or if < 10% of the wetland was found to be flooded upon arrival.

We began the area search survey at the location where we first arrived at the wetland unit. We noted the start time and began walking along the perimeter of the wetland unit. Using binoculars and spotting scopes, we counted all waterbirds within the wetland unit's boundary. Only waterbirds observed on the water, on the ground, or flying low (< 100 m) over the water were considered to be associated with the wetland unit. Observations of birds flying high (> 100 m) over the wetland unit and in transit were excluded. We always attempted to identify each observation to species; however, if that was not possible, we identified observations to the lowest taxonomic level possible (e.g., unidentified dabbling duck, gull, or sandpiper).

Two‐point counts, located at a minimum of 400 m from one another (Conway 2011), were performed at each wetland unit. At some very small wetlands, a single point count was performed. Point count locations were randomized along the wetland perimeter using the ‘Create Random Points’ tool in ArcMap (ArcMap 10.6.1; Environmental Systems Research Institute, Redlands, California). As the observer doing an area search survey came upon a point count location while walking along the wetland unit perimeter, they paused the current survey and began a 10‐min point count survey following the Standardized North American Marsh Bird Monitoring Protocol (Conway 2011). Point counts were separated into a 3‐min passive phase, in which the observer listened and looked for birds, and an active phase during which calls of secretive marsh bird species were broadcast on a portable 21‐watt Bluetooth speaker. Species calls obtained from the Standardized North American Marsh Bird Monitoring Protocol were broadcast sequentially, with 30 s of silence between species, and included black rail ( *Laterallus jamaicensis* ), least bittern (*Botaurus exilis*), sora ( *Porzana carolina* ), Virginia rail ( *Rallus limicola* ), American bittern ( *Botaurus lentiginosus* ), common gallinule (*Gallinula galeata*), and pied‐billed grebe ( *Podilymbus podiceps* ).

### 2.3 Habitat Assessment

After completing the bird survey, we performed a habitat assessment of the wetland unit either later on the same day (99% of all surveys) or within 6 days of the bird survey. First, we performed an overall, large‐scale assessment by visually estimating the percent of the flooded area of the wetland unit with emergent vegetation (% emergent vegetation), no emergent vegetation (% open water), and bare or vegetated mudflats (% mud). We also estimated the percent of the flooded area of the wetland unit that comprised tall and dense emergent vegetation stands of hardstem bulrush ( *Schoenoplectus acutus* ), cattails (*Typha* spp.), and common reed ( *Phragmites australis* ; % tall vegetation). We counted all trees and utility poles within the wetland unit and within a 50 m buffer outward from the wetland unit boundary and summed these to obtain an index of the number of available perch sites for avian predators. For wetlands that were adjacent to dense tree lines, we estimated the number of trees by counting the trees in a representative subsection and extrapolating to the entire tree area. Finally, we calculated a shape index of the flooded area of each wetland unit at the time it was surveyed, based on the polygon of the water's edge, as:

$$\text{Shape index}=\frac{0.25\times \text{perimeter}\;\left(m\right)}{\sqrt{\text{area}\;\left(m^{2}\right)}}$$

following McGarigal (2014), where a smaller shape index denoted a more rounded flooded wetland area and a larger shape index denoted a more elongated and meandering flooded wetland area with more shoreline relative to wetland area.

Second, we performed a small‐scale assessment using line transects within each of the wetland units to measure emergent vegetation height and water depth. We randomized the transect starting point by returning to the point where we began the earlier area search bird survey and used a random number table to determine the direction (clockwise or counterclockwise along the wetland perimeter from the area search starting point) and distance (100–200 m) to start the transect. All transects began at the water's edge and then moved perpendicularly from the shoreline into the wetland unit. Beginning at the water's edge, we measured water depth and average emergent vegetation height (average height above the water surface of all the vegetation within a 1‐m radius) at 2‐m intervals until we reached a distance of 10 m from the water's edge. This enabled us to estimate shoreline depth and slope (within 10 m of the shore). We then continued to measure water depth and average emergent vegetation height at 10‐m intervals out to 200 m from the water's edge (walking perpendicular to the shoreline). At this point, we turned 90° (direction chosen at random) and continued to measure water depth and average emergent vegetation height at 10‐m intervals for another 100 m (now walking parallel to the shoreline). Finally, we turned 90° back toward the shoreline where we began and measured water depth and average emergent vegetation height over 10‐m intervals for 200 m until we reached the shoreline (walking perpendicular to the shoreline again). For the final 10 m from the shoreline, we again collected data at 2‐m intervals to estimate shoreline depth and slope. This procedure enabled us to collect approximately 50–70 (mean ± SD: 58 ± 12) sample points for each wetland habitat survey. For some wetlands that were smaller, we reduced the 10‐m intervals to 5‐m intervals to ensure we obtained a large number of transect sample points. Moreover, at a few wetlands that were drying up and where the flooded area had receded into narrow channels, we instead performed strip transects (straight lines across the narrow, flooded area at 2‐m intervals), spaced approximately 50 m apart.

From these habitat transects, we obtained estimates for the average vegetation height and average water depth for each wetland unit survey using transect samples collected > 10 m from the shoreline. We calculated the coefficient of variation (CV) for water depth (water depth CV) and emergent vegetation height (vegetation height CV) for each wetland unit survey as the standard deviation of each metric divided by its mean. We also estimated shoreline slope using transect samples collected ≤ 10 m from the water's edge as follows:

$$\text{Shoreline slope}=\frac{\left(\text{Depth}\;\left(\mathrm{cm}\right)\;10\;m\;\text{from shore}-\text{Depth}\left(\mathrm{cm}\right)\;\mathrm{at}\;\text{shore}\right)}{10\;\left(m\right)}$$

### 2.4 Total Flooded Area

We estimated the total flooded area within each wetland unit during each survey using a handheld global positioning system (GPS; Garmin GPSMAP 64st, Garmin Ltd.) to track our path as we walked along the flooded wetland perimeter during the bird survey. If we were unable to walk close to the water's edge during part or all of the survey, we instead approximated the water's edge by marking the extent of the flooded area on preprinted maps of the wetland unit and using these notes to later digitize the water's edge in ArcMap. We then converted GPS tracks, or the digitized lines of the wetland water's edge, to polygons and calculated the total flooded area of each wetland unit during each survey using the Calculate Geometry tool in ArcMap.

### 2.5 Surrounding Land Cover

We evaluated the influence of surrounding land cover classes on bird density and diversity using the 2022 and 2023 U.S. Department of Agriculture (USDA)‐National Agricultural Statistics Service (NASS) Cropland Data Layers (USDA NASS 2024). The Cropland Data Layer (USDA NASS 2024) is a national, georeferenced raster dataset derived from satellite imagery (Landsat 8 and 9, ESA Sentinel 2A and 2B) collected during the growing season (spring through fall) with a spatial resolution of 30 m that aggregates land cover into 85 standardized categories that include agricultural crop types as well as other land cover types (e.g., wetlands, developed lands). We combined the Cropland Data Layer land cover classes into the following four broad categories (Table 1): agricultural land (rice, alfalfa/hay, clover/grass/vetch, fallow, orchards, and row crops), developed lands (low, medium, and high intensity), orchards, uplands, and wetlands (herbaceous wetlands, woody wetlands, and open water), and examined the influence of these surrounding land cover categories on bird use of wetlands enrolled in the California Waterfowl Habitat Program. Land cover categories were not mutually exclusive. For example, uplands included some agricultural lands, and orchards were a subset of agricultural lands. For each wetland surveyed, we calculated the number of hectares of each land cover category within 5 km of the wetland unit's boundary. We used a 5 km buffer following Conlisk et al. (2023) because landscape features at this scale have been found to influence waterbird use of wetland habitats in the Central Valley (Elphick 2008) and because this represents common daily movements of wintering dabbling ducks (McDuie et al. 2019). In addition, we calculated the distance from each wetland unit's boundary to the nearest ≥ 10‐ha contiguous patch of agricultural lands, orchards, uplands, and developed lands (we did not evaluate the distance to the nearest wetland patch because there was little variation in this measurement among wetland units). We then examined the influence of the natural log of the amount of, and the natural log of the distance to, surrounding land cover categories on bird use of wetlands enrolled in the California Waterfowl Habitat Program. We used natural log transformations for these variables because they spanned multiple orders of magnitude, and we hypothesized that these effects would be nonlinear. Because the distance of some wetland units to some land cover categories was sometimes zero, we added half of the minimum non‐zero distance to all datapoints before the natural log transformation. We similarly added half of the minimum non‐zero number of perch sites (0.5) to all datapoints to account for wetlands with no surrounding perch sites and then natural log transformed the number of perch sites before analysis.

**TABLE 1:** Cropland Data Layer cover classes (USDA NASS 2024) comprised each of the land cover categories examined for their potential influence on bird use of wetlands enrolled in the California Waterfowl Habitat Program.

Land cover category	Cropland land cover classes
Agricultural lands	Alfalfa, hay, clover/wildflowers/vetch, fallow/idle, orchards, rice, row crops, sod/grass seed
Developed lands	Developed low, medium, and high intensity
Orchards	Almonds, apples, apricots, avocados, cherries, citrus, grapes, olives, other tree crops, peaches, pears, pecans, pistachios, plums, pomegranates, prunes, walnuts
Uplands	Alfalfa, barley, chickpeas, clover/wildflowers, double crop barley/corn, double crop oats/corn, double crop triticale/corn, double crop winter wheat/corn, double crop winter wheat/sorghum, dry beans, durum wheat, fallow/idle, grassland/pasture, oats, other hay, rye, shrubland, sod/grass seed, spring wheat, triticale, winter wheat
Wetlands	Herbaceous wetlands, woody wetlands, open water

We note that the amount of wetland habitat, as well as all other land cover categories we examined, was based on USDA NASS Cropland Data Layers that are collected using satellite imagery during the spring through fall. As a result, our analysis did not examine the amount of flooded wetland habitat available within 5 km of a wetland unit at the time of the survey; rather, it examined the overall amount of the wetland habitat land cover category for the year and therefore may have included surrounding wetland habitat that was not actually flooded at the time of the survey. Nevertheless, because wetlands are most likely to be flooded during the wetter winter and early spring, we believe our results largely reflect winter and early spring bird densities on California Waterfowl Habitat Program wetlands as a function of the amount of surrounding flooded wetland habitat available.

### 2.6 Proximity to Potential Upland Nesting Habitat

We quantified the amount of upland habitat within a 1.3 km buffer from each surveyed wetland boundary. We chose a 1.3 km buffer because this represents the median distance traveled by dabbling duck broods at 2 weeks after hatch from upland nesting habitat to nearby wetlands (S.H. Peterson, USGS unpublished data); thus, it would reflect upland habitat from which the surveyed wetland may be accessible to young duckling broods. We also quantified the distance from each wetland boundary to the nearest ≥ 1 ha contiguous patch of upland habitat. We designated potential upland habitat using the Cropland Data Layer cover classes in Table 1, which included grasslands, compatible agricultural lands, and pasture.

### 2.7 Experimental Design and Statistical Analyses

We investigated factors influencing use of California Waterfowl Habitat Program wetlands by specific waterbird guilds at critical times during which these wetlands are available to birds. First, we evaluated bird use during late winter and spring migration using surveys conducted between February 3 and April 20. For this time period we examined (1) all waterbird density, (2) dabbling duck density, (3) shorebird density, (4) wading bird density, and (5) species richness (number of species). We included 205 wood ducks ( *Aix sponsa* ; 0.4% of the 55,539 ducks used in the dabbling duck analyses) in our evaluation of dabbling duck density because they breed locally in the Central Valley and are an important species for management. Second, we examined (6) the density of secretive marsh birds during the spring using surveys conducted between April 3 and May 31, which coincides with the time period recommended by the Standardized North American Marsh Bird Monitoring Protocol for these species in California's Central Valley (Conway 2011). Finally, we examined (7) the density of ducks during the breeding season for those species that nest locally in the Central Valley and Suisun–Delta (mallard, gadwall, cinnamon teal, and wood ducks) using surveys conducted between April 3 and July 28.

For each of the 7 guilds above, we conducted separate analyses in the program R v. 4.2.2 (Bates et al. 2015; R Core Team 2022) to evaluate the influence of various spatial–temporal and environmental variables obtained from our habitat assessment and surrounding land cover (Table 2). For each analysis, we included the study year and the natural log of the area of flooded habitat, ln(flooded area), as base variables in all models. Because surveyed wetland units varied by size, and because wetland units were not always flooded all the way to their boundaries, we included the natural log of the flooded area as an offset term in order to account statistically for the overall amount of flooded habitat present during each survey; thus, the dependent variable could be interpreted as bird density. In addition, in all analyses except spring secretive marsh bird density, we included wetland unit as a random effect to account for any repeated sampling of wetland units. There was no within‐year repeat sampling of specific wetland units for the spring secretive marsh bird analysis (April 3–May 31), and therefore, a random effect of wetland unit was unnecessary. We then built a balanced set of all combinations of the categorical variable region and linear trends for the remaining variables in Table 2, with the exception that any variables that were highly correlated (*r* ≥ 0.7) were not allowed to be in the same model. We also explored potential quadratic relationships for date, average water depth, average vegetation height, and % emergent vegetation after centering each variable on its median value, separately for each analysis, to reduce multicollinearity between the linear and quadratic terms. To avoid over‐parameterization, we limited models to a maximum of 6 fixed effects, including the base variables study year and ln(flooded area); thus, up to 4 additional fixed effects were allowed to enter each model (*n* = 6703 total models). However, for the wading bird density analysis, we had relatively fewer surveys where these birds were detected, and the low number of detections resulted in non‐convergence for some models. Therefore, we did not consider quadratic terms for date, average water depth, average vegetation height, and % emergent vegetation (*n* = 5897 models).

**TABLE 2:** Variables used to evaluate bird use of wetlands enrolled in the California Waterfowl Habitat Program in the Sacramento Valley, San Joaquin Valley, and Suisun–Delta regions of California during 2002 and 2023.

Variable	Description
Spatial–temporal	
Date	Recorded as the number of days since January 1
Minutes after sunrise	Number of minutes between sunrise on the survey date and the start time of the survey; used to account for potential differences in bird detection with survey start time
Region	Sacramento Valley, San Joaquin Valley, or Suisun–Delta
Study year	Year 1 (2022) or Year 2 (2023)
Large‐scale habitat	
ln(flooded area)	Flooded area (ha) at the time of the survey
Flooded shape index	Index based on the ratio of perimeter to area of the flooded wetland area; larger values indicate longer, narrower wetlands
Percent emergent vegetation	Percent of the flooded area with emergent vegetation present
Percent tall vegetation	Percent of the flooded area with tall, dense stands of hardstem bulrush, cattails, or common reed
Percent mud	Percent of the flooded area composed of mudflat habitat
ln(number of perch sites)	Number of trees and utility poles within 50 m of the wetland boundary
Small‐scale habitat	
Shoreline slope	Water depth change (cm/m) from the water's edge to approximately 10 m from the water's edge
Average water depth	Average depth of water (cm) across all transect sample points ≥ 10 m from the shoreline; included as both linear and quadratic effects
Water depth CV	Coefficient of variation for the average depth across all transect sample points ≥ 10 m from the shoreline
Average vegetation height	Average height of emergent vegetation (cm) across all transect sample points ≥ 10 m from the shoreline
Vegetation height CV	Coefficient of variation for the average height of emergent vegetation across all transect sample points
Surrounding land cover	
ln(distance to nearest agricultural land)	Distance (m) from the wetland boundary to the nearest contiguous block of agricultural land ≥ 10 ha
ln(distance to nearest developed land)	Distance (m) from the wetland boundary to the nearest contiguous block of developed land ≥ 10 ha
ln(distance to nearest orchard)	Distance (m) from the wetland boundary to the nearest contiguous block of orchards ≥ 10 ha
ln(distance to nearest upland)	Distance (m) from the wetland boundary to the nearest contiguous block of upland habitat ≥ 1 ha
ln(agricultural ha within 5 km)	Hectares of agricultural land within 5 km of the wetland boundary
ln(orchard ha within 5 km)	Hectares of orchards within 5 km of the wetland boundary
ln(upland ha within 1.3 km)	Hectares of upland habitat within 1.3 km of the wetland boundary
ln(upland ha within 5 km)	Hectares of upland habitat within 5 km of the wetland boundary
ln(wetland ha within 5 km)	Hectares of wetlands within 5 km of the wetland boundary

For all density analyses, we used generalized linear models with a second‐order negative binomial distribution and a log link (glmmTMB function from the R package glmmTMB; Brooks et al. 2017), which is most appropriate for analyses of count data when the variance increases non‐linearly with the mean. For the species richness analysis, we used generalized linear models with a Poisson distribution and a log link.

For each analysis, we ranked models according to maximum parsimony using an information‐theoretic approach and second‐order Akaike's Information Criterion (AIC_c_), in which the difference in AIC_c_ values (ΔAIC_c_) between the best model (lowest AIC_c_) and each other model was used to assign model rank (Burnham and Anderson 2002). We present Akaike model weights (*w*_i_), or the relative likelihood of each model given all the models in the candidate set, and evidence ratios, or the relative weight of support between models derived by dividing the weight of the top model by the weight of the model of interest. Furthermore, by comparing the evidence ratio of the top model to the same model with one of the variables removed, we evaluated the relative importance of each variable present in the top model. Finally, we calculated the adjusted relative importance of each variable by summing the weight of all models in the candidate set that included a particular variable after accounting for the fact that some variables occurred in more models than other variables (Ackerman et al. 2015). Variables with an adjusted relative importance > 1.0 were considered to be very important; variables with an adjusted relative importance of 0–1.0 were considered to be somewhat important; and variables with a score < 0 were considered to be unimportant.

We present predictions for the effects of important variables on bird density and species richness. To account for model selection uncertainty, we calculated model‐averaged predictions based on models comprising 90% of the cumulative model weights. We predicted values for each model using the model‐specific coefficients, weighted these predicted values by their model weight, and then averaged these weighted predicted values to obtain model‐averaged predictions. For each time period and each variable of interest, we predicted between the 5th and 95th quantiles of the observed values while holding all other continuous variables at their median value (Table 3) and setting the study year to ‘year 1’ (2022) and the region to ‘San Joaquin Valley’.

**TABLE 3:** Median values used in predictions of bird density and species richness in wetlands enrolled in the California Waterfowl Habitat Program in the Sacramento Valley, San Joaquin Valley, and Suisun–Delta regions of California during 2022 and 2023.

Variable	February 3–April 20^a^	April 3–May 31^b^	April 3–July 28^c^
Spatial–temporal			
Date (days since January 1)^d^	81 (34–110)	108 (93–151)	123 (93–209)
Minutes after sunrise	65 (0–256)	95 (11–245)	92 (0–245)
Region	San Joaquin Valley	San Joaquin Valley	San Joaquin Valley
Study year	Year 1 (2022)	Year 1 (2022)	Year 1 (2022)
Large‐scale habitat			
Flooded area (ha)	12.9 (1.1–166.3)	10.0 (0.4–48)	9.9 (0.4–48)
Flooded shape index	1.25 (0.98–3.74)	1.35 (1.01–6.28)	1.40 (0.97–6.28)
Percent emergent vegetation^d^	41 (3.4–91.8)	42 (0–100)	50 (0–100)
Percent tall vegetation (bulrush, cattails, or common reed)	19 (0–71)	22 (0–100)	25 (0–100)
Percent mud	11 (0–69)	15 (0–50)	10 (0–60)
Number of perch sites	11 (0–1500)	14 (0–815)	18 (0–1002)
Small‐scale habitat			
Shoreline slope	1.25 (0.05–8.09)	1.17 (0.05–7.51)	1.29 (0.05–8.32)
Average water depth (cm)^d^	22.8 (4.70–108.89)	23.8 (3.9–75.1)	27.6 (3.9–104.4)
Water depth CV	0.43 (0.05–1.18)	0.42 (0.05–1.18)	0.39 (0.05–1.18)
Average vegetation height (cm)^d^	18.1 (0–129.9)	18.7 (0–147.6)	23.8 (0–176.4)
Vegetation height CV	1.56 (0–7.14)	1.70 (0–5.39)	1.51 (0–6.16)
Surrounding land cover			
Distance (m) to nearest agricultural land	1354 (5–7647)	1484 (5–7647)	1559 (5–7647)
Distance (m) to nearest developed land	7299 (3245‐12,559)	7296 (3245‐12,559)	7373 (3245‐12,559)
Distance (m) to nearest orchard	3935 (33.9–15,163)	4019 (158–15,555)	4101 (158–15,555)
Distance (m) to nearest upland	171 (10–2096)	202 (10–2208)	194 (10–2208)
Agricultural ha within 5 km	3131 (174–8970)	3087 (159–8970)	3073 (159–8970)
Orchard ha within 5 km	455 (22–3016)	436 (20–3016)	404 (20–3016)
Upland ha within 1.3 km	NA	NA	82 (3–707)
Upland ha within 5 km	1607 (453–6766)	1799 (531–6766)	NA
Wetland ha within 5 km	4963 (179–8301)	5028 (179–8018)	5056 (179–8018)

*Note:* Data ranges are in parentheses.

^a^ For predictions related to all waterbirds, dabbling ducks, shorebirds, wading birds, and species richness.

^b^ For predictions related to secretive marsh birds.

^c^ For predictions related to breeding ducks.

^d^ Variable was centered, separately for each time period, by subtracting the median from each value.

### 2.8 Seasonal Versus Semi‐Permanent Wetlands

We modeled bird density (separately for all waterbirds, dabbling ducks, shorebirds, and wading birds) and species richness between February 3 and April 20 as a function of the wetland type (seasonal wetland vs. semi‐permanent wetland) in the previous year. For this analysis, we only included wetlands for which we knew (1) the wetland type during the year of the survey, (2) the wetland type during the previous year, and (3) the wetland type did not change between years. We excluded two surveys in which the wetland type in the previous year was unknown and six surveys in which the wetland type changed between years. The resulting dataset, therefore, included wetland units that were managed consistently in the year of and the year prior to the survey.

We performed two analyses to examine the effect of wetland type on bird density and diversity. In the first analysis, in addition to wetland type, we included the spatial–temporal variables region, minutes after sunrise, and date, as well as all the surrounding land cover variables in Table 2. We did not include any wetland unit‐specific habitat variables (e.g., water depth, percent emergent vegetation cover, vegetation height) because these were related to the wetland type and therefore may obscure detection of an effect of wetland type. In the second analysis, we included all variables in Table 2, including wetland unit‐specific habitat variables. This second analysis allowed us to examine if wetland type was an important predictor of winter and early spring bird use of California Waterfowl Habitat Program wetlands even when competing with variables accounting for differences in measured wetland features such as water depth, percent emergent vegetation cover, and vegetation height. We again built balanced sets of all combinations of up to six non‐correlated (*r* ≥ 0.7) fixed effects. We used generalized linear models with a second‐order negative binomial distribution and a log link (glmmTMB function from the R package glmmTMB) for all density analyses and a Poisson distribution and a log link for species richness. For each analysis, we ranked models according to maximum parsimony using an information‐theoretic approach and AIC_c_ as described above. Finally, we examined if wetland habitat variables differed between seasonal and semi‐permanent wetlands using a generalized linear model with a binomial distribution and a logit link (glmmTMB function from the R package glmmTMB). We evaluated a single model with the binary response variable (seasonal vs. semi‐permanent wetland) and predictor wetland habitat variables water depth, average vegetation height, percent emergent vegetation cover, and percent cover of tall, dense stands of hardstem bulrush, cattails, and/or common reed (% tall vegetation). In all models, we included study year and the natural log of the area of flooded habitat as fixed effects as well as wetland unit as a random effect to account for any repeated sampling of wetland units.

## 3 Results

We conducted 240 surveys of 117 individual wetlands (61 seasonal wetlands, 54 semi‐permanent wetlands, and 2 wetlands that were either semi‐permanent wetlands or reverse‐cycle wetlands depending on the year) on 36 properties enrolled in the California Waterfowl Habitat Program during February–June of 2022 and February–July of 2023 (Figure 1). We recorded a total of 158,842 waterbirds belonging to 67 species; the most abundant birds were dabbling ducks (55,539 [205 were wood ducks]; 35.0% of all waterbirds), followed by geese (50,264; 31.6%), American coots ( *Fulica americana* , 24,163; 15.2%), shorebirds (22,239; 14.0%), wading birds (3450; 2.2%), diving ducks (1864; 1.2%), and secretive marsh birds (840; 0.5%). Although we observed 50,264 geese overall, 32,687 (65%) were snow geese ( *Anser caerulescens* ) observed during a single wetland survey in the Sacramento Valley in March of 2022. Mallard was the most frequently observed species among surveys (165 surveys), followed by American coot (146 surveys), northern shoveler (*Spatula clypeata*; 138 surveys), killdeer ( *Charadrius vociferus* ; 107 surveys), and green‐winged teal ( *Anas crecca* ; 107 surveys).

### 3.1 All Waterbird Taxa: Late Winter and Early Spring

We observed 154,491 waterbirds during 159 of the 160 surveys (99%) that were conducted between February 3 and April 20. The top model for waterbird density included the percent of tall, dense stands of hardstem bulrush, cattails, and/or common reed (% tall vegetation); the natural log of wetland ha within 5 km; region; and water depth CV (Table 4). No other model was competitive (ΔAIC_c_ ≤ 2.0). Relative variable importance scores indicated strong support for the effect of % tall vegetation (5.25), the natural log of wetland ha within 5 km (4.89), region (4.23), and water depth CV (1.14; Table 5).

**TABLE 4:** Model selection results for waterbird density within wetlands enrolled in the California Waterfowl Habitat Program in the Sacramento Valley, San Joaquin Valley, and Suisun–Delta regions of California during February 3–April 20 of 2022 (Year 1) and 2023 (Year 2).

Model (base model: study year + ln(flooded area) + wetland unit)	*k*	−2 LogL	AIC_c_	ΔAIC_c_	*w*_i_	Evidence ratio
**+ % tall vegetation + ln(wetland ha within 5 km) + region + water depth CV**	**9**	**2298.17**	**2317.37**	**0.00**	**0.38**	**1.00**
+ % tall vegetation + ln(wetland ha within 5 km) + region + average vegetation height	9	2300.29	2319.49	2.11	0.13	2.88
+ % tall vegetation + ln(wetland ha within 5 km) + region + shoreline slope	9	2300.74	2319.94	2.57	0.10	3.61
+ % tall vegetation + ln(wetland ha within 5 km) + region + flooded shape index	9	2301.94	2321.14	3.77	0.06	6.58
**+ % tall vegetation + ln(wetland ha within 5 km) + region**	**8**	**2304.70**	**2321.66**	**4.28**	**0.04**	**8.51**
+ % tall vegetation + ln(wetland ha within 5 km) + region + date	9	2302.69	2321.89	4.52	0.04	9.58
+ % tall vegetation + ln(wetland ha within 5 km) + region + minutes after sunrise	9	2303.33	2322.53	5.16	0.03	13.17
+ % tall vegetation + ln(wetland ha within 5 km) + region + % mud	9	2303.62	2322.82	5.45	0.02	15.22
+ % tall vegetation + ln(wetland ha within 5 km) + region + ln(number of perches)	9	2303.74	2322.94	5.57	0.02	16.21
+ % tall vegetation + ln(wetland ha within 5 km) + region + % emergent vegetation	9	2303.91	2323.11	5.74	0.02	17.60
+ % tall vegetation + ln(wetland ha within 5 km) + region + water depth	9	2304.10	2323.30	5.93	0.02	19.35
+ % tall vegetation + ln(wetland ha within 5 km) + region + ln(distance to nearest developed land)	9	2304.26	2323.46	6.09	0.02	20.97
+ % tall vegetation + ln(wetland ha within 5 km) + region + vegetation height CV	9	2304.48	2323.68	6.31	0.02	23.43
**+ % tall vegetation + region + water depth CV**	**8**	**2316.71**	**2333.67**	**16.29**	**0.00**	**3452.61**
**+ ln(wetland ha within 5 km) + % tall vegetation + water depth CV**	**7**	**2321.88**	**2336.62**	**19.25**	**0.00**	**1.51 × 10** ^**4**^
**+ region + ln(wetland ha within 5 km) + water depth CV**	**8**	**2328.70**	**2345.66**	**28.28**	**0.00**	**1.39 × 10** ^**6**^
*Base model: study year + ln(flooded area) + wetland unit*	4	2344.40	2352.66	35.29	0.00	8.18 × 10^9^

*Note:* All models (*n* = 6703) included the variables from the base model (study year + ln(flooded area)) and the random effect (wetland unit). The top models that represented 0.90 cumulative model weights, the null (base) model, and models similar to the top model but with one of the variables removed (bolded text) are presented.

Abbreviations: −2 LogL, −2 × log(likelihood); AIC_c_, second‐order Akaike information criterion; Evidence ratio, weight of evidence that the model with the lowest AIC_c_ value is better than the current model; *k*, number of parameters in the model; *w*_i_, Akaike model weight or the likelihood of the current model given the data, relative to other models in the candidate set; ΔAIC_c_, difference in AIC_c_ between the top model and the current model.

**TABLE 5:** Relative importance of variables examined for their influence on bird density and species richness for wetlands enrolled in the California Waterfowl Habitat Program in the Sacramento Valley, San Joaquin Valley, and Suisun–Delta regions of California during 2022 and 2023.

Variable	February 3–April 20					April 3–May 31	April 3–July 28
	Waterbird density	Dabbling duck density	Shorebird density	Wading bird density	Waterbird species richness	Secretive marsh bird density	Breeding duck density
Spatial–temporal							
Date	−1.74	2.02	−1.68	−1.43	−0.56	4.06	6.50
Date^2^	−4.42	2.37	−3.13	NA	−1.72	0.71	0.11
Minutes after sunrise	−1.94	−2.25	−1.92	1.00	−1.21	−2.09	−2.07
Region	4.23	−2.61	−1.59	−0.39	−1.51	−1.90	1.95
Large‐scale habitat							
Flooded shape index	−1.15	−1.54	−2.21	−2.08	9.15	−1.98	−1.96
Percent emergent vegetation	−2.37	0.09	0.16	−1.85	−0.55	−1.77	−2.19
Percent emergent vegetation^2^	−5.89	−1.89	−1.22	NA	−1.27	−2.22	−4.29
Percent tall vegetation (bulrush, cattail, common reed)	5.25	−0.45	−1.75	−0.56	1.56	3.14	−1.76
Percent mud	−1.87	−0.60	−1.70	4.67	0.41	−1.53	−2.04
Number of perch sites	−1.61	−2.03	1.92	2.52	−0.74	−2.01	6.08
Small‐scale habitat							
Shoreline slope	−0.35	−0.94	−2.21	−1.96	0.37	−0.39	−1.28
Water depth	−1.97	5.26	12.52	−1.52	0.99	3.01	−2.11
Water depth^2^	−4.29	1.49	5.32	NA	1.55	0.03	−4.79
Water depth CV	1.14	0.47	−0.40	−2.04	−1.07	−1.94	−1.66
Average vegetation height	−0.31	1.75	0.59	−1.93	−1.20	−0.22	−0.56
Average vegetation height^2^	−3.07	−0.91	−1.60	NA	−0.93	−2.04	−3.19
Vegetation height CV	−2.45	−1.98	−0.85	−1.30	−1.23	−1.31	−1.05
Surrounding land cover							
Distance to nearest ag land	−3.82	−1.93	−1.51	−0.46	−0.95	0.11	−1.35
Distance to nearest developed land	−2.03	−1.14	−0.64	−1.43	−1.26	−1.75	−1.85
Distance to nearest orchard	−3.75	−1.50	−1.66	−0.39	−0.62	−0.29	−1.13
Distance to nearest upland	−2.46	−1.25	−2.15	1.49	−1.19	−1.68	0.63
Agricultural ha within 5 km	−3.72	−1.55	−1.15	−0.67	−0.73	−1.11	−0.58
Orchard ha within 5 km	−312	−1.66	−1.81	0.31	−0.85	−1.08	−0.38
Upland ha within 1.3 km	NA	NA	NA	NA	NA	NA	0.06
Upland ha within 5 km	−3.51	−1.24	−1.29	−1.82	−1.08	−1.52	NA
Wetland ha within 5 km	4.89	−1.02	0.02	0.67	−0.07	0.39	−0.65

*Note:* Positive numbers ≥ 1.0 indicate variables with a strong influence (dark blue, dark red), positive numbers 0.0–1.0 indicate variables with less influence (light blue, light red), and negative numbers indicate variables with no influence. Red indicates a negative relationship; blue indicates a positive relationship. Gray indicates a class variable with a strong influence.

**FIGURE 2:**
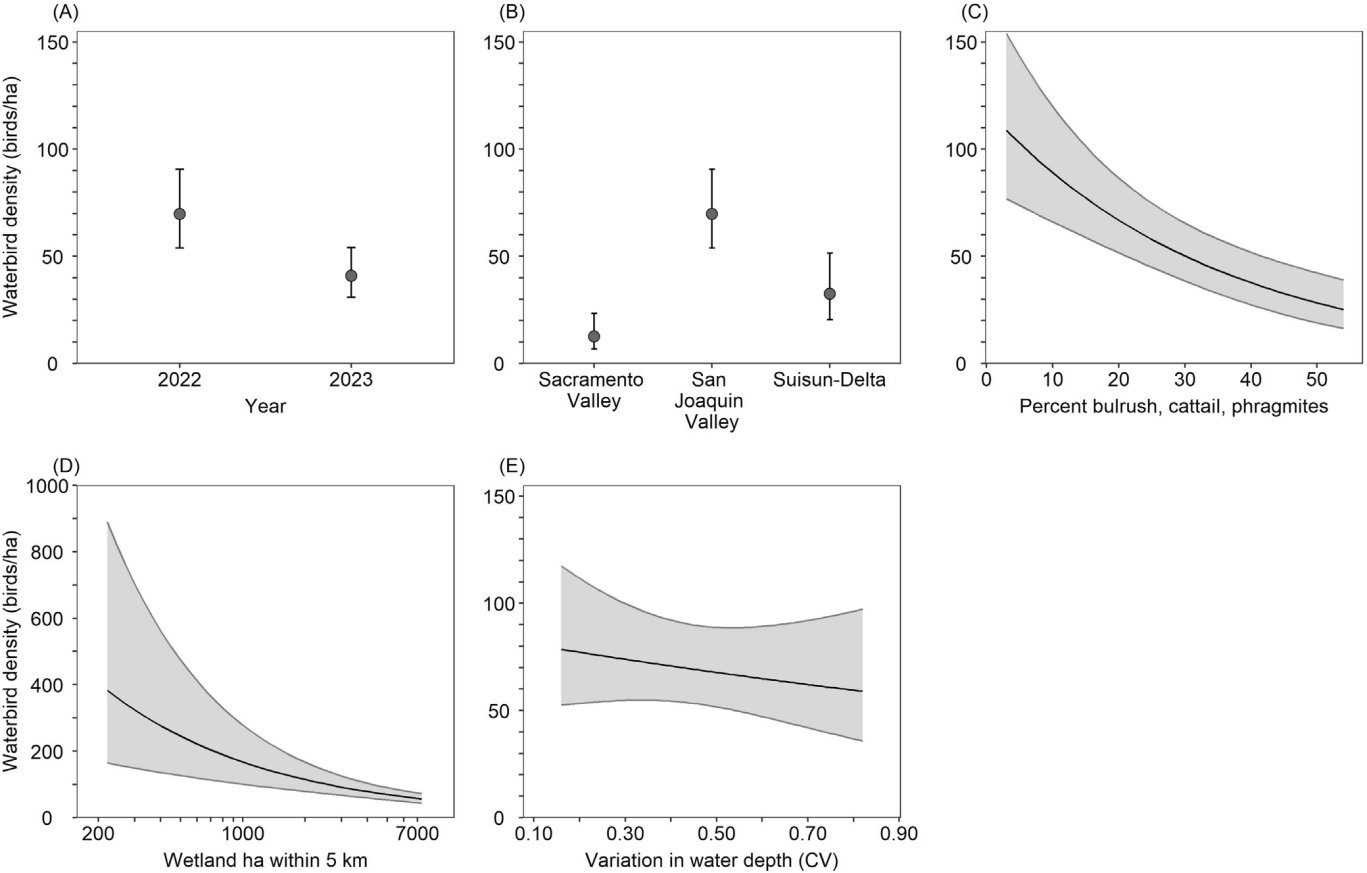
Model‐averaged predictions (±95% confidence intervals) for density of all waterbirds in wetlands enrolled in the California Waterfowl Habitat Program during February 3–April 20 as a function of various habitat variables. Predictions (A) and (C–E) were made for the San Joaquin Valley region, by holding all non‐focal continuous variables at their median value (Table 3). Predictions (B–E) were made for the year 2022.

Model‐averaged predictions indicated that between February 3 and April 20, California Waterfowl Habitat Program wetlands supported 41% fewer birds in 2023 than in 2022, and bird density was greatest in the San Joaquin Valley (2022: 69.8 waterbirds/ha, 2023: 40.8 waterbirds/ha), followed by the Suisun–Delta (2022: 32.4 birds/ha, 2023: 19.0 birds/ha) and the Sacramento Valley (2022: 12.6 birds/ha, 2023: 7.4 birds/ha). Waterbird density decreased 77% from 111.9 birds/ha in wetlands with 2% tall vegetation cover to 25.3 birds/ha in wetlands with 54% tall vegetation cover; decreased 86% from an average of 382.6 birds/ha in wetlands with 213 ha of other wetland habitats within 5 km of the wetland boundary to 53.4 birds/ha in wetlands with 7830 ha of other surrounding wetland habitats; and decreased 25% from 78.5 birds/ha in wetlands with water depth CV of 0.16 to 59.0 birds/ha in wetlands with water depth CV of 0.82 (Figure 2).

### 3.2 Dabbling Ducks: Late Winter and Early Spring

We observed 53,766 dabbling ducks during 155 of the 160 surveys (97%) conducted between February 3 and April 20. The top model for dabbling duck density included the average water depth, a quadratic effect of date, and average vegetation height (Table 6). A second model that included water depth CV also was competitive (ΔAIC_c_ = 1.57, Table 6). Relative variable importance scores indicated strong support for the effects of water depth (5.26 linear form, 1.49 quadratic form), date (2.02 linear form, 2.37 quadratic form), and average vegetation height (1.75 linear form) on dabbling duck density, with less support for water depth CV (0.47) and % emergent vegetation (0.09; Table 5).

**TABLE 6:** Model selection results for dabbling duck density within wetlands enrolled in the California Waterfowl Habitat Program in the Sacramento Valley, San Joaquin Valley, and Suisun–Delta regions of California during February 3–April 20 of 2022 (Year 1) and 2023 (Year 2).

Model (base model: study year + ln(flooded area) + wetland unit)	*k*	−2 LogL	AIC_c_	ΔAIC_c_	*w*_i_	Evidence ratio
**+ water depth + date + date** ^**2**^ **+ average vegetation height**	**8**	**1982.33**	**1999.29**	**0.00**	**0.11**	**1.00**
+ water depth + date + average vegetation height + water depth CV	8	1983.90	2000.85	1.57	0.05	2.19
+ water depth + date + date^2^ + % emergent vegetation	8	1984.83	2001.78	2.50	0.03	3.48
+ water depth + date + date^2^ + water depth CV	8	1985.03	2001.99	2.70	0.03	3.86
+ water depth + water depth^2^ + date + average vegetation height	8	1985.07	2002.02	2.74	0.03	3.93
+ water depth + date + date^2^ + % tall vegetation	8	1985.23	2002.18	2.90	0.03	4.25
+ water depth + date + average vegetation height + % mud	8	1985.59	2002.54	3.26	0.02	5.09
+ water depth + date + average vegetation height + % emergent vegetation	8	1985.90	2002.85	3.57	0.02	5.96
+ water depth + date + average vegetation height + ln(distance to nearest developed land)	8	1986.26	2003.21	3.93	0.02	7.13
+ water depth + date + water depth CV + % emergent vegetation	8	1986.32	2003.27	3.99	0.02	7.35
**+ water depth + date + average vegetation height**	**7**	**1988.61**	**2003.34**	**4.06**	**0.02**	**7.60**
**+ water depth + date + date** ^**2**^	**7**	**1991.63**	**2006.37**	**7.08**	**0.00**	**34.55**
**+ water depth + average vegetation height**	**6**	**1997.13**	**2009.68**	**10.40**	**0.00**	**181.07**
**+ date + date** ^**2**^ **+ average vegetation height**	**7**	**2003.55**	**2018.29**	**19.01**	**0.00**	**1.34 × 10** ^**4**^
*Base model: study year + ln(flooded area) + wetland unit*	4	2023.96	2032.21	32.93	0.00	1.41 × 10^7^

*Note:* All models (*n* = 6703) included the variables from the base model (study year + ln(flooded area)) and the random effect (wetland unit). All models with ΔAICc ≤ 4.0 from the top model, the null (base) model, and models similar to the top model but with one of the variables removed (bolded text) are presented.

Abbreviations: −2 LogL, −2 × log(likelihood); AIC_c_, second‐order Akaike information criterion; evidence ratio, weight of evidence that the model with the lowest AIC_c_ value is better than the current model; *k*, number of parameters in the model; *w*_i_, Akaike model weight or the likelihood of the current model given the data, relative to other models in the candidate set; ΔAIC_c_, difference in AIC_c_ between the top model and the current model.

**FIGURE 3:**
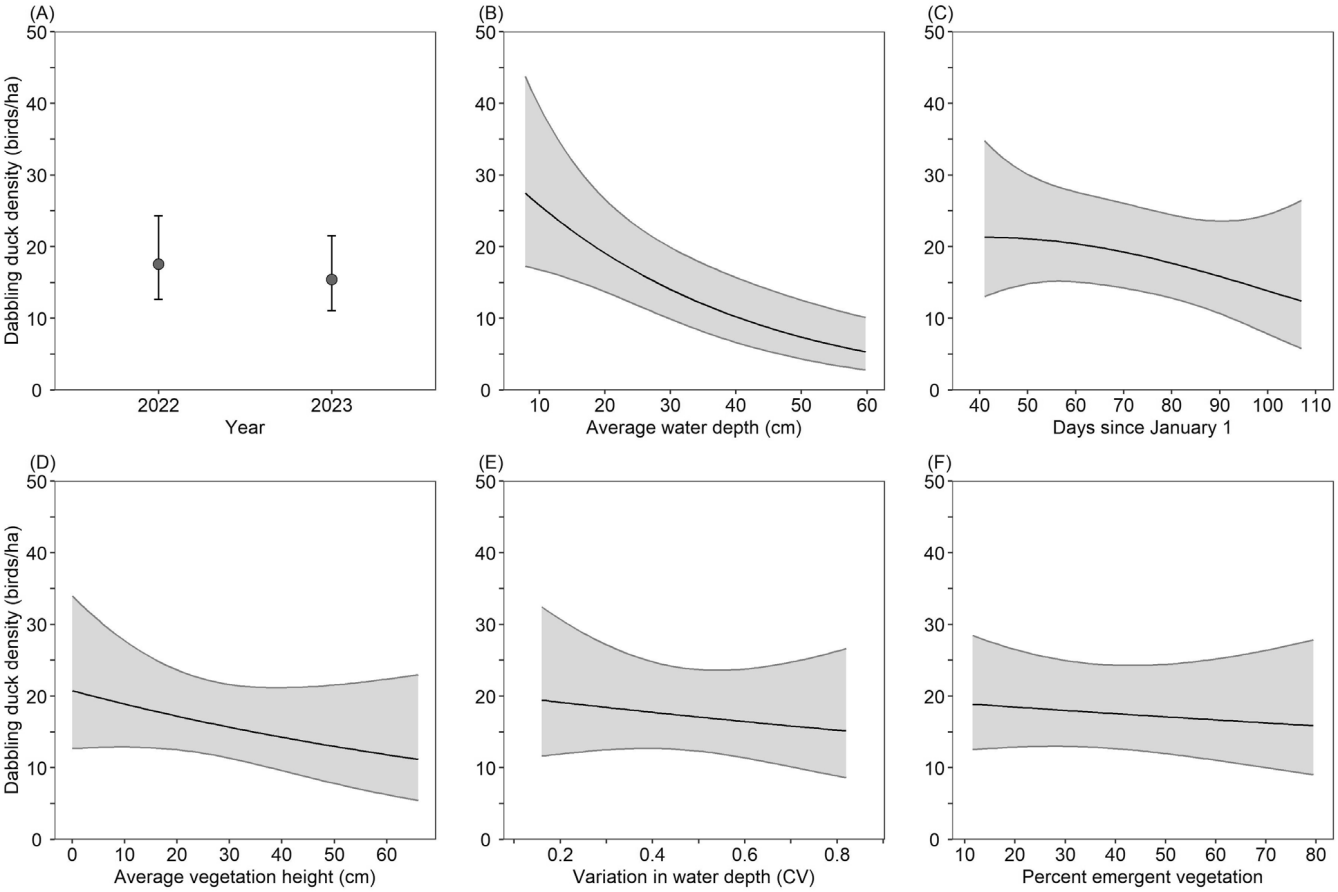
Model‐averaged predictions (±95% confidence intervals) for dabbling duck density in wetlands enrolled in the California Waterfowl Habitat Program during February 3–April 20 as a function of various habitat variables. Predictions (A–F) were made for the San Joaquin Valley region by holding all non‐focal continuous variables at their median value (Table 3). Predictions (B–F) were made for the year 2022.

Model‐averaged predictions indicated that between February 3 and April 20, California Waterfowl Habitat Program wetlands supported similar dabbling duck densities in 2022 (17.5 dabbling ducks/ha) and 2023 (15.4 dabbling ducks/ha), and density did not differ among regions. Dabbling duck density decreased 81% from 28.3 dabbling ducks/ha in wetlands with an average water depth of 7 cm to only 5.3 dabbling ducks/ha in wetlands with an average water depth of 60 cm; decreased 42% from 21.3 dabbling ducks/ha on February 10 (day 41) to 12.4 ducks/ha on April 17 (day 107); decreased 46% from 20.6 dabbling ducks/ha in wetlands with an average vegetation height of 1 cm to 11.2 dabbling ducks/ha in wetlands with an average vegetation height of 66 cm; decreased 22% from 19.4 dabbling ducks/ha in wetlands with water depth CV of 0.16 to 15.1 dabbling ducks/ha in wetlands with water depth CV of 0.82; and decreased 16% from 18.9 dabbling ducks/ha in wetlands with 11% emergent vegetation cover to 15.9 dabbling ducks/ha in wetlands with 80% emergent vegetation cover (Figure 3).

### 3.3 Shorebirds: Late Winter and Early Spring

We observed 21,227 shorebirds during 136 of the 160 surveys (85%) conducted between February 3 and April 20. The top model for shorebird density included a quadratic effect for water depth, the natural log of the number of perch sites, and average vegetation height (Table 7). Models that included the % emergent vegetation, water depth CV, and the natural log of the wetland ha within 5 km also were competitive (ΔAIC_c_ = 0.28–1.75, Table 7). Relative variable importance scores indicated strong support for the effects of water depth (12.52 linear form, 5.32 quadratic form) and the natural log of the number of perch sites (1.92), with less support for the average vegetation height (0.59), the % emergent vegetation cover (0.16), and the natural log of the wetland ha within 5 km (0.02; Table 5). However, the 85% CI for the conditional model‐averaged slope coefficient for wetland ha within 5 km overlapped zero, suggesting that the amount of wetland ha within 5 km of the wetland boundary was an uninformative parameter that did not influence shorebird density (Arnold 2010).

**TABLE 7:** Model selection results for shorebird density within wetlands enrolled in the California Waterfowl Habitat Program in the Sacramento Valley, San Joaquin Valley, and Suisun–Delta regions of California during February 3–April 20 of 2022 (Year 1) and 2023 (Year 2).

Model (base model: study year + ln(flooded area) + wetland unit)	*k*	−2LogL	AIC_c_	ΔAIC_c_	*w*_i_	Evidence ratio
**+ water depth + water depth** ^**2**^ **+ ln(number of perch sites) + average vegetation height**	**8**	**1486.39**	**1503.34**	**0.00**	**0.12**	**1.00**
+ water depth + water depth^2^ + ln(number of perch sites) + % emergent vegetation	8	1486.66	1503.62	0.28	0.10	1.15
+ water depth + water depth^2^ + ln(number of perch sites) + water depth CV	8	1487.40	1504.35	1.01	0.07	1.66
+ water depth + water depth^2^ + ln(number of perch sites) + ln(wetland ha within 5 km)	8	1488.14	1505.09	1.75	0.05	2.39
+ water depth + water depth^2^ + ln(number of perch sites) + vegetation height CV	8	1488.58	1505.53	2.19	0.04	2.99
**+ water depth + water depth** ^**2**^ **+ ln(number of perch sites)**	**7**	**1491.78**	**1506.52**	**3.18**	**0.02**	**4.90**
+ water depth + water depth^2^ + average vegetation height + ln(distance to nearest developed land)	8	1489.62	1506.57	3.23	0.02	5.03
+ water depth + water depth^2^ + % emergent vegetation + ln(distance to nearest developed land)	8	1490.57	1507.53	4.18	0.01	8.10
+ water depth + water depth^2^ + ln(number of perch sites) + date	8	1490.65	1507.60	4.26	4.26	4.26
+ water depth + water depth^2^ + ln(wetland ha within 5 km) + region	9	1488.53	1507.73	4.39	0.01	8.97
+ water depth + water depth^2^ + ln(number of perch sites) + % tall vegetation	8	1490.79	1507.75	4.40	0.01	9.04
+ water depth + water depth^2^ + ln(number of perch sites) + ln(upland ha within 5 km)	8	1491.04	1507.99	4.65	0.01	10.23
+ water depth + water depth^2^ + average vegetation height + % emergent vegetation	8	1491.13	1508.08	4.74	0.01	10.69
**+ water depth + water depth** ^**2**^ **+ average vegetation height**	**7**	**1493.72**	**1508.46**	**5.11**	**0.01**	**12.90**
**+ water depth + average vegetation height + ln(number of perch sites)**	**7**	**1494.98**	**1509.72**	**6.37**	**0.00**	**24.21**
**+ average vegetation height + ln(number of perch sites)**	**6**	**1534.19**	**1546.74**	**43.40**	**0.00**	**2.65 × 10** ^**9**^
*Base model: study year + ln(flooded area) + wetland unit*	4	1548.86	1557.12	53.78	0.00	4.76 × 10^11^

*Note:* All models (*n* = 6703) included the variables from the base model (study year + ln(flooded area)) and the random effect (wetland unit). The top models that represented 0.50 cumulative model weights, the null (base) model, and models similar to the top model but with one of the variables removed (bolded text) are presented.

Abbreviations: −2LogL, −2 × log(likelihood); AIC_c_, second‐order Akaike information criterion; evidence ratio, weight of evidence that the model with the lowest AIC_c_ value is better than the current model; *k*, number of parameters in the model; *w*_i_, Akaike model weight or the likelihood of the current model given the data, relative to other models in the candidate set; ΔAIC_c_, difference in AIC_c_ between the top model and the current model.

**FIGURE 4:**
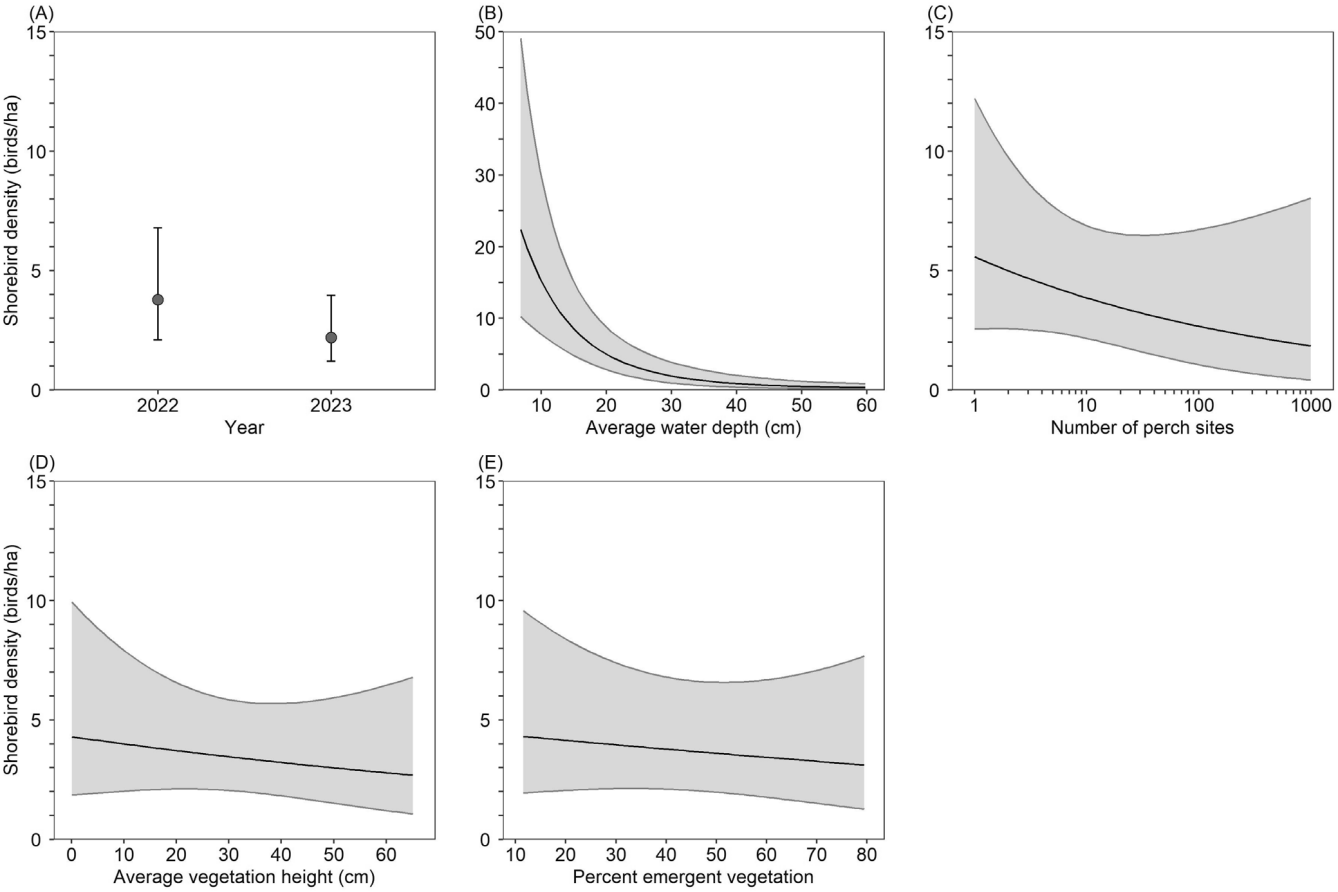
Model‐averaged predictions (±95% confidence intervals) for shorebird density in wetlands enrolled in the California Waterfowl Habitat Program during February 3–April 20 as a function of various habitat variables. Predictions (A–E) were made for the San Joaquin Valley region, by holding all non‐focal continuous variables at their median value (Table 3). Predictions (B–E) were made for the year 2022.

Model‐averaged predictions indicated that between February 3 and April 20, California Waterfowl Habitat Program wetlands supported 42% fewer shorebirds in 2023 (Sacramento Valley: 2.0 shorebirds/ha, San Joaquin Valley: 2.2 shorebirds/ha, Suisun–Delta: 2.2 shorebirds/ha) than in 2022 (Sacramento Valley: 3.5 shorebirds/ha, San Joaquin Valley: 3.8 birds/ha, Suisun–Delta: 3.7 shorebirds/ha), but shorebird density was similar among regions. Shorebird density decreased 99% from 22.4 shorebirds/ha in wetlands with a water depth of 7 cm to 0.3 shorebirds/ha in wetlands with a water depth of 60 cm; decreased 68% from 5.6 shorebirds/ha in wetlands with 1 perch site to 1.8 shorebirds/ha in wetlands with 1000 perch sites, decreased 37% from 4.3 shorebirds/ha in wetlands with an average vegetation height of 1 cm to 2.7 shorebirds/ha in wetlands with an average vegetation height of 66 cm, and decreased 28% from 4.3 shorebirds/ha in wetlands with 11% emergent vegetation cover to 3.1 shorebirds/ha in wetlands with 80% emergent vegetation cover (Figure 4).

### 3.4 Wading Birds: Late Winter and Early Spring

We observed 2741 wading birds during 99 of the 160 surveys (62%) conducted between February 3 and April 20. The top model for wading bird density included the % mud, the natural log of the number of perch sites, the natural log of the distance to the nearest upland habitat, and minutes after sunrise (Table 8). Models that included % tall vegetation, the natural log of orchard ha within 5 km, and the natural log of wetland ha within 5 km also were competitive (ΔAIC_c_ = 0.58–1.09, Table 8). Relative variable importance scores indicated strong support for the effects of % mud (4.67), the natural log of the number of perch sites (2.52), the natural log of the distance to nearest upland (1.49), and the minutes after sunrise (1.00), with less support for the natural log of the wetland ha within 5 km (0.67) and the natural log of the orchard ha within 5 km (0.31; Table 5).

**TABLE 8:** Model selection results for wading bird density within wetlands enrolled in the California Waterfowl Habitat Program in the Sacramento Valley, San Joaquin Valley, and Suisun–Delta regions of California during February 3–April 20 of 2022 (Year 1) and 2023 (Year 2).

Model (base model: study year + ln(flooded area) + wetland unit)	*k*	−2LogL	AIC_c_	ΔAIC_c_	*w*_i_	Evidence ratio
**+ % mud + ln(number of perch sites) + ln(distance to nearest upland) + minutes after sunrise**	**8**	**895.45**	**912.41**	**0.00**	**0.08**	**1.00**
+ % mud + ln(number of perch sites) + minutes after sunrise + ln(orchard ha within 5 km)	8	896.03	912.98	0.58	0.06	1.34
+ % mud + ln(number of perch sites) + ln(distance to nearest upland) + % tall vegetation	8	896.45	913.40	1.00	0.05	1.65
+ % mud + ln(number of perch sites) + minutes after sunrise + ln(wetland ha within 5 km)	8	896.54	913.49	1.09	0.05	1.72
**+ % mud + ln(number of perch sites) + ln(distance to nearest upland)**	**7**	**900.12**	**914.86**	**2.45**	**0.02**	**3.41**
+ % mud + ln(number of perch sites) + ln(distance to nearest upland) + region	9	895.71	914.91	2.50	0.02	3.50
+ % mud + ln(number of perch sites) + ln(distance to nearest upland) + ln(orchard ha within 5 km)	8	898.00	914.95	2.54	0.02	3.57
+ % mud + ln(number of perch sites) + ln(distance to nearest upland) + vegetation height CV	8	898.13	915.08	2.67	0.02	3.81
+ % mud + ln(distance to nearest upland) + minutes after sunrise + region	9	895.90	915.10	2.70	0.02	3.85
+ % mud + ln(number of perch sites) + ln(distance to nearest upland) + ln(distance to nearest orchard)	8	898.25	915.20	2.79	0.02	4.04
+ % mud + ln(number of perch sites) + minutes after sunrise + ln(distance to nearest orchard)	8	898.63	915.58	3.17	0.02	4.89
+ % mud + ln(number of perch sites) + ln(distance to nearest upland) + ln(wetland ha within 5 km)	8	898.76	915.71	3.31	0.02	5.22
+ % mud + ln(number of perch sites) + minutes after sunrise + ln(agricultural ha within 5 km)	8	899.01	915.96	3.55	0.01	5.91
+ % mud + ln(distance to nearest upland) + minutes after sunrise + ln(wetland ha within 5 km)	8	899.01	915.97	3.56	0.01	5.93
+ % mud + ln(number of perch sites) + minutes after sunrise + ln(distance to nearest agricultural land)	8	899.17	916.12	3.72	0.01	6.41
+ % mud + ln(number of perch sites) + ln(distance to nearest upland) + ln(distance to nearest agricultural land)	8	899.25	916.21	3.80	0.01	6.69
+ % mud + ln(number of perch sites) + ln(distance to nearest upland) + water depth	8	899.30	916.25	3.84	0.01	6.84
+ % mud + ln(number of perch sites) + ln(distance to nearest upland) + date	8	899.40	916.35	3.95	0.01	7.20
+ % mud + ln(number of perch sites) + % tall vegetation + ln(orchard ha within 5 km)	8	899.41	916.36	3.95	0.01	7.22
+ % mud + minutes after sunrise + ln(wetland ha within 5 km) + region	9	897.16	916.36	3.96	0.01	7.23
+ % mud + ln(number of perch sites) + ln(wetland ha within 5 km) + % tall vegetation	8	899.49	916.44	4.04	0.01	7.53
**+ % mud + ln(distance to nearest upland) + minutes after sunrise**	**7**	**903.06**	**917.80**	**5.39**	**0.01**	**14.81**
**+ % mud + ln(number of perch sites) + minutes after sunrise**	**7**	**906.23**	**920.97**	**8.56**	**0.00**	**72.31**
**+ ln(number of perch sites) + ln(distance to nearest upland) + minutes after sunrise**	**7**	**911.95**	**926.69**	**14.28**	**0.00**	**1263.49**
*Base model: study year + ln(flooded area) + wetland unit*	4	939.00	947.25	34.85	0.00	3.69 × 10^7^

*Note:* All models (*n* = 5897) included the variables from the base model (study year + ln(flooded area)) and the random effect (wetland unit). The top models that represented 0.50 cumulative model weights, the null (base) model, and models similar to the top model but with one of the variables removed (bolded text) are presented.

Abbreviations: −2LogL, −2 × log(likelihood); AIC_c_, second‐order Akaike information criterion; evidence ratio, weight of evidence that the model with the lowest AIC_c_ value is better than the current model; *k*, number of parameters in the model; *w*_i_, Akaike model weight or the likelihood of the current model given the data, relative to other models in the candidate set; ΔAIC_c_, difference in AIC_c_ between the top model and the current model.

**FIGURE 5:**
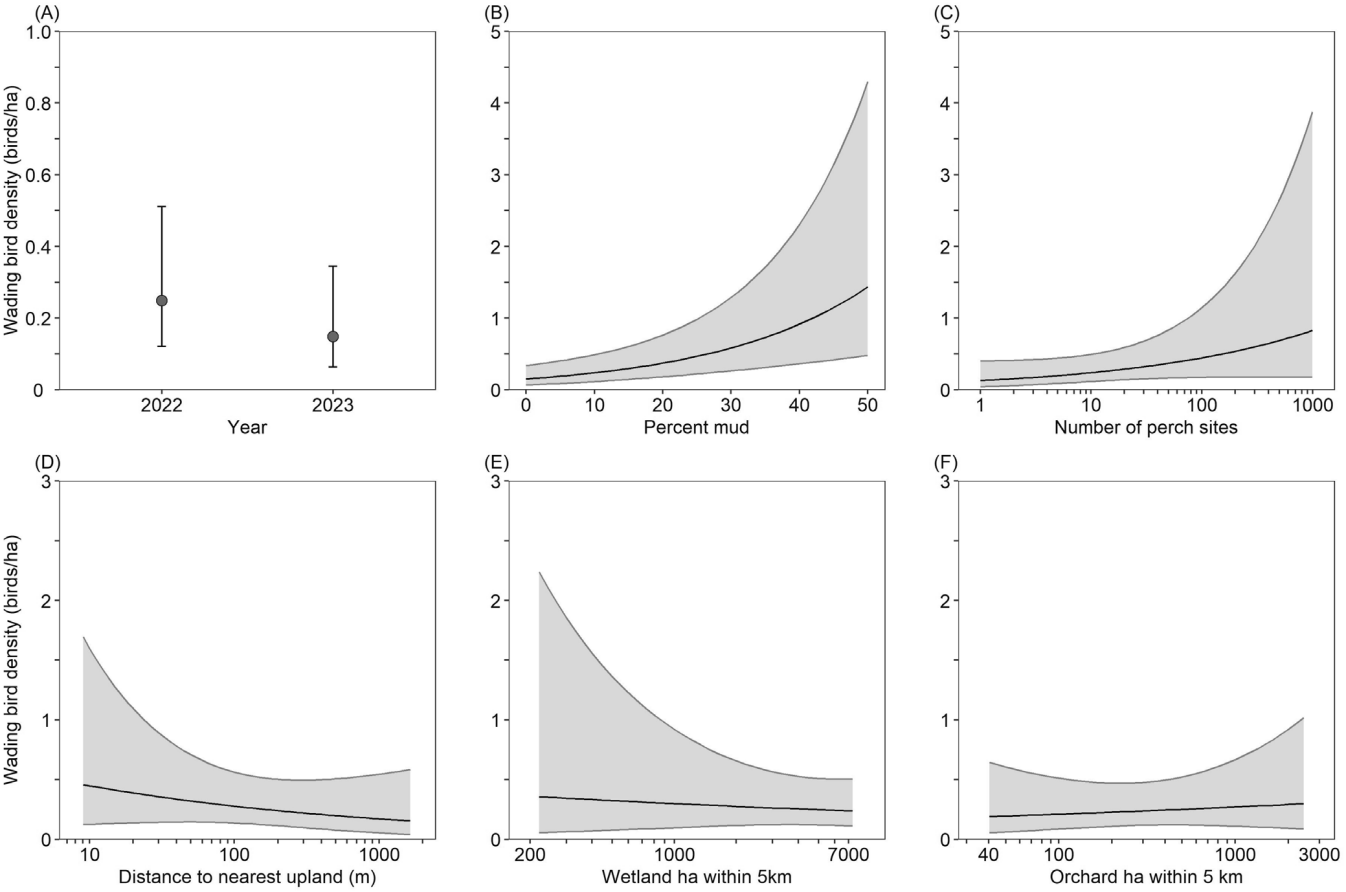
Model‐averaged predictions (±95% confidence intervals) for wading bird density in wetlands enrolled in the California Waterfowl Habitat Program during February 3–April 20 as a function of various habitat variables. Predictions (A–F) were made for the San Joaquin Valley region, by holding all non‐focal continuous variables at their median value (Table 3). Predictions (B–F) were made for the year 2022.

Model‐averaged predictions indicated that between February 3 and April 20, California Waterfowl Habitat Program wetlands supported more wading birds in 2022 (Sacramento Valley: 0.27 wading birds/ha, San Joaquin Valley: 0.25 wading birds/ha, Suisun–Delta: 0.21 wading birds/ha) than in 2023 (Sacramento Valley: 0.16 wading birds/ha, San Joaquin Valley: 0.15 wading birds/ha, Suisun–Delta: 13 wading birds/ha), but were similar among regions. However, the 85% CI for the conditional model‐averaged slope coefficient for study year overlapped zero, suggesting that wading bird density did not vary substantially between years. Wading bird density increased 813% from 0.15 wading birds/ha in wetlands with 0% mud cover to 1.37 wading birds/ha in wetlands with 49% mud cover; increased 538% from 0.13 wading birds/ha in wetlands with 1 perch site to 0.83 wading birds/ha in wetlands with 1000 perch sites, decreased 64% from 0.45 wading birds/ha in wetlands 10 m from the nearest upland habitat to 0.16 wading birds/ha in wetlands 1693 m from the nearest upland, decreased 33% from 0.36 wading birds/ha in wetlands with 213 ha of wetlands within 5 km to 0.24 wading birds/ha in wetlands with 7830 ha of surrounding wetlands, and increased 58% from 0.19 wading birds/ha in wetlands with 37 ha of orchards within 5 km to 0.30 wading birds/ha in wetlands with 2640 ha of surrounding orchard (Figure 5).

### 3.5 Waterbird Species Richness: Late Winter and Early Spring

We observed 63 waterbird species during surveys conducted between February 3 and April 20. The top model for species richness included the flooded shape index, the percent of tall, dense stands of hardstem bulrush, cattails, or common reed (% tall vegetation), and a quadratic effect of water depth (Table 9). No other model was competitive (ΔAIC_c_ ≤ 2.0). Relative variable importance scores indicated strong support for the effects of flooded shape index (9.15), % tall vegetation (1.56), and water depth (0.99 linear form, 1.55 quadratic form), with less support for the percent mud (0.41) and shoreline slope (0.37; Table 5).

**TABLE 9:** Model selection results for waterbird species richness within wetlands enrolled in the California Waterfowl Habitat Program in the Sacramento Valley, San Joaquin Valley, and Suisun–Delta regions of California during February 3–April 20 of 2022 (Year 1) and 2023 (Year 2).

Model (base model: study year + ln(flooded area) + wetland unit)	*k*	−2LogL	AIC_c_	ΔAIC_c_	*w*_i_	Evidence ratio
**+ flooded shape index + % tall vegetation + water depth + water depth** ^**2**^	**7**	**1020.79**	**1035.53**	**0.00**	**0.07**	**1.00**
+ flooded shape index + % tall vegetation + % mud + shoreline slope	7	1023.28	1038.02	2.49	0.02	3.48
**+ flooded shape index + % tall vegetation + water depth**	**6**	**1025.61**	**1038.16**	**2.63**	**0.02**	**3.73**
+ flooded shape index + % tall vegetation + water depth + % mud	7	1024.06	1038.79	3.27	0.01	5.12
+ flooded shape index + % tall vegetation + % mud	6	1026.42	1038.97	3.44	0.01	5.59
+ flooded shape index + % tall vegetation + shoreline slope	6	1026.50	1039.05	3.52	0.01	5.82
+ flooded shape index + % tall vegetation + water depth + date	7	1024.34	1039.08	3.55	0.01	5.90
+ flooded shape index + % tall vegetation + shoreline slope + date	7	1024.45	1039.19	3.67	0.01	6.25
+ flooded shape index + % tall vegetation + water depth + shoreline slope	7	1024.59	1039.33	3.81	0.01	6.71
+ flooded shape index + % tall vegetation + water depth + ln(wetland ha within 5 km)	7	1024.75	1039.49	3.96	0.01	7.26
**+ flooded shape index + water depth + water depth** ^**2**^	**6**	**1027.70**	**1040.25**	**4.73**	**0.01**	**10.63**
**+ flooded shape index + % tall vegetation**	**5**	**1031.51**	**1041.90**	**6.38**	**0.00**	**24.25**
**+ % tall vegetation + water depth + water depth** ^**2**^	**6**	**1042.42**	**1054.97**	**19.45**	**0.00**	**1.67 × 10** ^**4**^
*Base model: study year + ln(flooded area) + wetland unit*	3	1065.98	1072.14	36.61	0.00	8.91 × 10^7^

*Note:* All models (*n* = 6703) included the variables from the base model (study year + ln(flooded area)) and the random effect (wetland unit). All models with ΔAICc ≤ 4.0 from the top model, the null (base) model, and models similar to the top model but with one of the variables removed (bolded text) are presented.

Abbreviations: −2LogL, −2 × log(likelihood); AIC_c_, second‐order Akaike information criterion; evidence ratio, weight of evidence that the model with the lowest AIC_c_ value is better than the current model; *k*, number of parameters in the model; *w*_i_, Akaike model weight or the likelihood of the current model given the data, relative to other models in the candidate set; ΔAIC_c_, difference in AIC_c_ between the top model and the current model.

**FIGURE 6:**
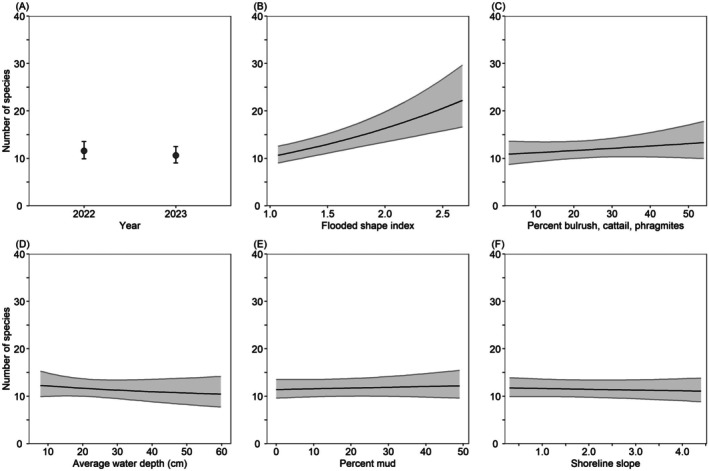
Model‐averaged predictions (±95% confidence intervals) for waterbird species richness in wetlands enrolled in the California Waterfowl Habitat Program during February 3–April 20 as a function of various habitat variables. All predictions (A–E) were made for the San Joaquin Valley region, by holding all non‐focal continuous variables at their median value (Table 3). Predictions (B–E) were made for the year 2022.

Model‐averaged predictions indicated that between February 3 and April 20, California Waterfowl Habitat Program wetlands in the San Joaquin Valley supported 9% fewer species in 2023 (10.6) than in 2022 (11.6) and did not vary among regions. Species richness increased 118% from 10.2 species in wetlands with a flooded shape index of 1.0 (more rounded wetland) to 22.2 species in wetlands with a flooded shape index of 2.7 (elongated wetlands with greater perimeter to area); increased 23% from 10.8 species in wetlands with 2% tall vegetation cover to 13.3 species in wetlands with 54% tall vegetation cover, decreased 15% from 12.4 species in wetlands with a water depth of 7 cm to 10.5 species in wetlands with a water depth of 60 cm, increased 7% from 11.4 species in wetlands with 0% mud cover to 12.2 species in wetlands with 49% mud cover, and decreased 5% from 11.7 species in wetlands with a shoreline slope of 0.25 cm/m to 11.1 species in wetlands with a shoreline slope of 4.5 cm/m (Figure 6).

### 3.6 Secretive Marsh Birds

We observed 332 secretive marsh birds during 57 of the 82 surveys (70%) conducted between April 3 and May 31. The top model for secretive marsh bird density included the percent of tall, dense stands of hardstem bulrush, cattails, or common reed (% tall vegetation); date; water depth; and the distance to the nearest agricultural land (Table 10). Models that included the natural log of the wetland ha within 5 km and the natural log of the distance to the nearest orchards also were competitive (ΔAIC_c_ = 0.29–1.28, Table 10). Relative variable importance scores indicated strong support for the effects of date (4.06 linear form, 0.71 quadratic form), % tall vegetation (3.14), and water depth (3.01 linear form, 0.03 quadratic form), with less support for the natural log of the wetland ha within 5 km (0.39) and the natural log of the distance to nearest agricultural land (0.11; Table 5).

**TABLE 10:** Model selection results for secretive marsh bird density within wetlands enrolled in the California Waterfowl Habitat Program in the Sacramento Valley, San Joaquin Valley, and Suisun–Delta regions of California during April 1–May 31 of 2022 (Year 1) and 2023 (Year 2).

Model (base model: study year + ln(flooded area))	*k*	−2LogL	AIC_c_	ΔAIC_c_	*w*_i_	Evidence ratio
**+ % tall vegetation + water depth + date + ln(distance to nearest agricultural land)**	**7**	**358.74**	**374.25**	**0.00**	**0.11**	**1.00**
+ % tall vegetation + water depth + date + ln(wetland ha within 5 km)	7	359.03	374.54	0.29	0.09	1.15
+ % tall vegetation + water depth + date + ln(distance to nearest orchard)	7	360.02	375.54	1.28	0.06	1.90
**+ % tall vegetation + water depth + date**	**6**	**362.76**	**375.88**	**1.63**	**0.05**	**2.26**
+ % tall vegetation + water depth + date + date^2^	7	360.69	376.20	1.95	0.04	2.65
+ % tall vegetation + water depth + date + vegetation height CV	7	360.77	376.28	2.03	0.04	2.76
+ % tall vegetation + water depth + date + ln(orchard ha within 5 km)	7	361.17	376.68	2.42	0.03	3.36
+ % tall vegetation + water depth + water depth^2^ + date	7	361.67	377.19	2.93	0.02	4.34
+ % tall vegetation + water depth + date + ln(upland ha within 5 km)	7	361.83	377.35	3.09	0.02	4.69
+ % tall vegetation + water depth + date + average vegetation height	7	361.84	377.36	3.10	0.02	4.72
+ % tall vegetation + water depth + date + shoreline slope	7	361.90	377.41	3.16	0.02	4.85
**+ % tall vegetation + water depth + ln(distance to nearest agricultural land)**	**6**	**371.52**	**384.64**	**10.38**	**0.00**	**179.89**
**+ % tall vegetation + date + ln(distance to nearest agricultural land)**	**6**	**373.25**	**386.37**	**12.11**	**0.00**	**426.78**
**+ water depth + date + ln(distance to nearest agricultural land)**	**6**	**375.75**	**388.87**	**14.61**	**0.00**	**1489.86**
*Base model: study year + ln(flooded area)*	3	396.50	402.81	28.56	0.00	1.59 × 10^6^

*Note:* All models (*n* = 6703) included the variables from the base model (study year + ln(flooded area)). The top models that represented 0.50 cumulative model weights, the null (base) model, and models similar to the top model but with one of the variables removed (bolded text) are presented.

Abbreviations: −2LogL, −2 × log(likelihood); AIC_c_, second‐order Akaike information criterion; evidence ratio, weight of evidence that the model with the lowest AIC_c_ value is better than the current model; *k*, number of parameters in the model; *w*_i_, Akaike model weight or the likelihood of the current model given the data, relative to other models in the candidate set; ΔAIC_c_, difference in AIC_c_ between the top model and the current model.

**FIGURE 7:**
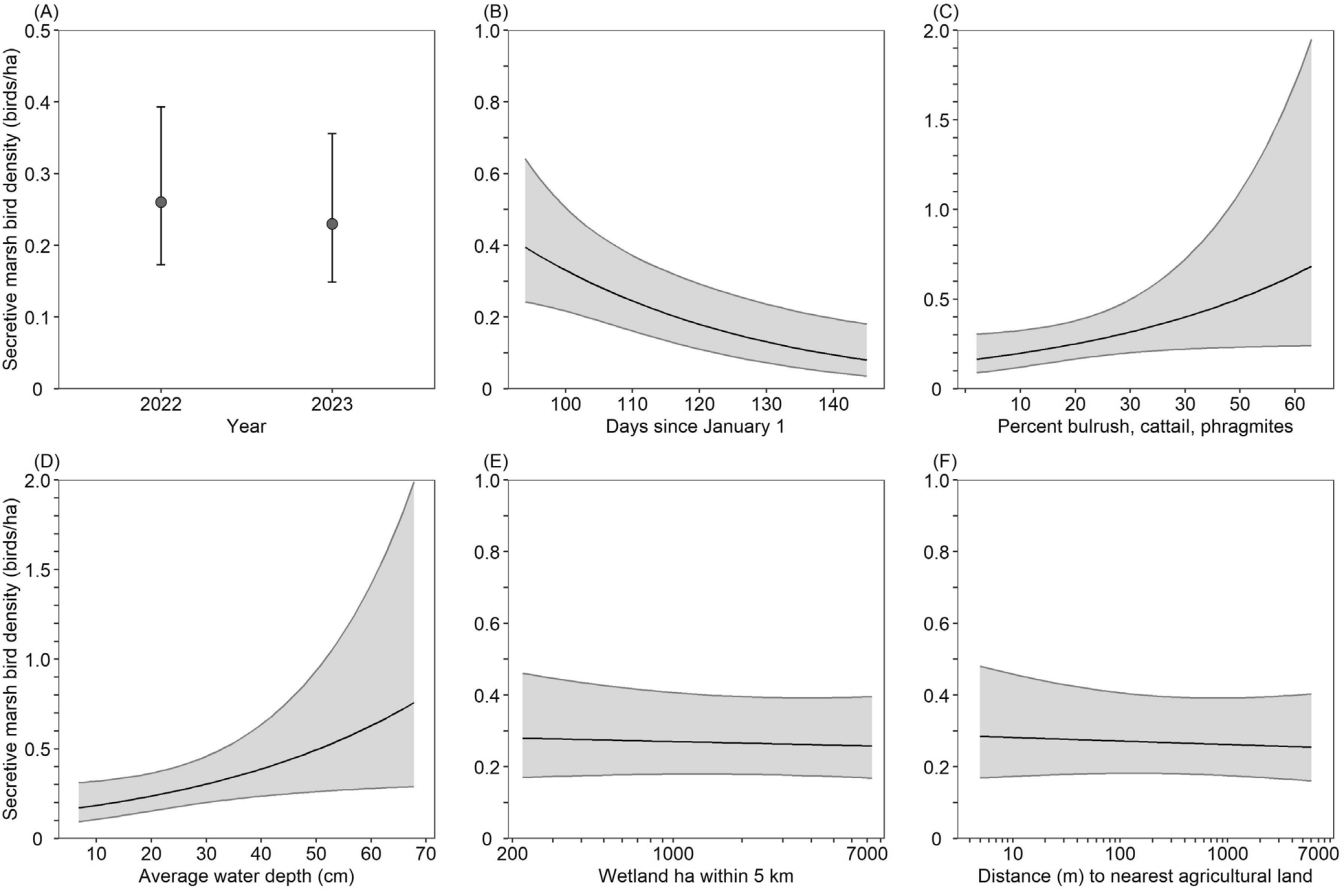
Model‐averaged predictions (±95% confidence intervals) for secretive marsh bird density in wetlands enrolled in the California Waterfowl Habitat Program during April 3–May 31 as a function of various habitat variables. Predictions (A–F) were made for the San Joaquin Valley region, by holding all non‐focal continuous variables at their median value (Table 3). Predictions (B–F) were made for the year 2022.

Model‐averaged predictions indicated that between April 3 and May 31, California Waterfowl Habitat Program wetlands in the San Joaquin Valley supported 0.26 secretive marsh birds per ha in 2022 and 0.23 secretive marsh birds per ha in 2023 but were similar among regions. However, the 85% CI for the conditional model‐averaged coefficient for study year overlapped zero, suggesting that secretive marsh bird density did not vary substantially by year. Secretive marsh bird density decreased 79% from 0.39 secretive marsh birds/ha on April 4 (day 94) to 0.08 secretive marsh birds/ha on May 25 (day 145); increased 312% from 0.17 secretive marsh birds/ha in wetlands with 2% tall vegetation cover to 0.70 secretive marsh birds/ha in wetlands with 64% tall vegetation cover, increased 347% from 0.17 secretive marsh birds/ha in wetlands with water depth of 6 cm to 0.76 secretive marsh birds/ha in wetlands with water depth of 68 cm, decreased 7% from 0.28 secretive marsh birds/ha in wetlands with 203 ha of wetlands within 5 km to 0.26 secretive marsh birds/ha in wetlands with 7815 ha of surrounding wetlands, and decreased 11% from 0.28 secretive marsh birds/ha in wetlands 5 m from the nearest agricultural land to 0.25 secretive marsh birds/ha in wetlands 6072 m from the nearest agricultural land (Figure 7).

### 3.7 Breeding Ducks

We observed 2049 ducks that are common breeders in the Central Valley (mallard, gadwall, cinnamon teal, and wood duck) during 113 of the 128 surveys (88%) conducted between April 3 and July 28. The top model for breeding duck density included date, the natural log of the number of perch sites, the natural log of the distance to the nearest upland habitat, and region (Table 11). Models that included vegetation height CV, average vegetation height, and the natural log of upland ha within 1.3 km of the wetland boundary were competitive (ΔAIC_c_ = 1.31–1.47, Table 11). Relative variable importance scores indicated strong support for the effects of date (6.50 linear form, 0.11 quadratic form), the natural log of the number of perch sites (6.08), and region (1.95), with less support for the natural log of the distance to nearest upland habitat (0.63) and the natural log of upland ha within 1.3 km (0.06; Table 5).

**TABLE 11:** Model selection results for locally breeding duck density within wetlands enrolled in the California Waterfowl Habitat Program in the Sacramento Valley, San Joaquin Valley, and Suisun–Delta regions of California during April 3–July 28 of 2022 (Year 1) and 2023 (Year 2).

Model (base model: study year + ln(flooded area) + wetland unit)	*k*	−2LogL	AIC_c_	ΔAIC_c_	*w*_i_	Evidence ratio
**+ date + ln(number of perch sites) + region + ln(distance to nearest upland)**	**9**	**839.81**	**859.33**	**0.00**	**0.10**	**1.00**
**+ date + ln(number of perch sites) + region**	**8**	**843.43**	**860.64**	**1.31**	**0.05**	**1.92**
+ date + ln(number of perch sites) + region + vegetation height CV	9	841.12	860.65	1.31	0.05	1.93
+ date + ln(number of perch sites) + region + average vegetation height	9	841.16	860.69	1.35	0.05	1.96
+ date + ln(number of perch sites) + region + ln(upland ha within 1.3 km)	9	841.28	860.80	1.47	0.05	2.08
+ date + ln(number of perch sites) + ln(distance to nearest upland) + ln(orchard ha within 5 km)	8	844.24	861.45	2.11	0.04	2.88
+ date + ln(number of perch sites) + ln(upland ha within 1.3 km) + ln(orchard ha within 5 km)	8	844.33	861.54	2.20	0.03	3.01
+ date + ln(number of perch sites) + ln(distance to nearest upland) + ln(agricultural ha within 5 km)	8	844.41	861.62	2.28	0.03	3.13
+ date + ln(number of perch sites) + ln(distance to nearest upland) + average vegetation height	8	844.42	861.63	2.30	0.03	3.15
+ date + ln(number of perch sites) + region + shoreline slope	9	842.15	861.67	2.34	0.03	3.22
+ date + ln(number of perch sites) + region + ln(wetland ha within 5 km)	9	842.77	862.30	2.96	0.02	4.40
**+ date + ln(number of perch sites) + ln(distance to nearest upland)**	**7**	**849.14**	**864.08**	**4.74**	**0.01**	**10.72**
**+ date + region + ln(distance to nearest upland)**	**8**	**858.65**	**875.86**	**16.53**	**0.00**	**3881.36**
**+ ln(number of perch sites) + region + ln(distance to nearest upland)**	**8**	**863.40**	**880.61**	**21.27**	**0.00**	**4.17 × 10** ^**4**^
*Base model: study year + ln(flooded area) + wetland unit*	4	887.26	895.59	36.25	0.00	7.44 × 10^7^

*Note:* All models (*n* = 6703) included the variables from the base model (study year + ln(flooded area)) and the random effect (wetland unit). The top models that represented 0.50 cumulative model weights, the null (base) model, and models similar to the top model but with one of the variables removed (bolded text) are presented.

Abbreviations: −2LogL, −2 × log(likelihood); AIC_c_, second‐order Akaike information criterion; evidence ratio, weight of evidence that the model with the lowest AIC_c_ value is better than the current model; *k*, number of parameters in the model; *w*_i_, Akaike model weight or the likelihood of the current model given the data, relative to other models in the candidate set; ΔAIC_c_, difference in AIC_c_ between the top model and the current model.

**FIGURE 8:**
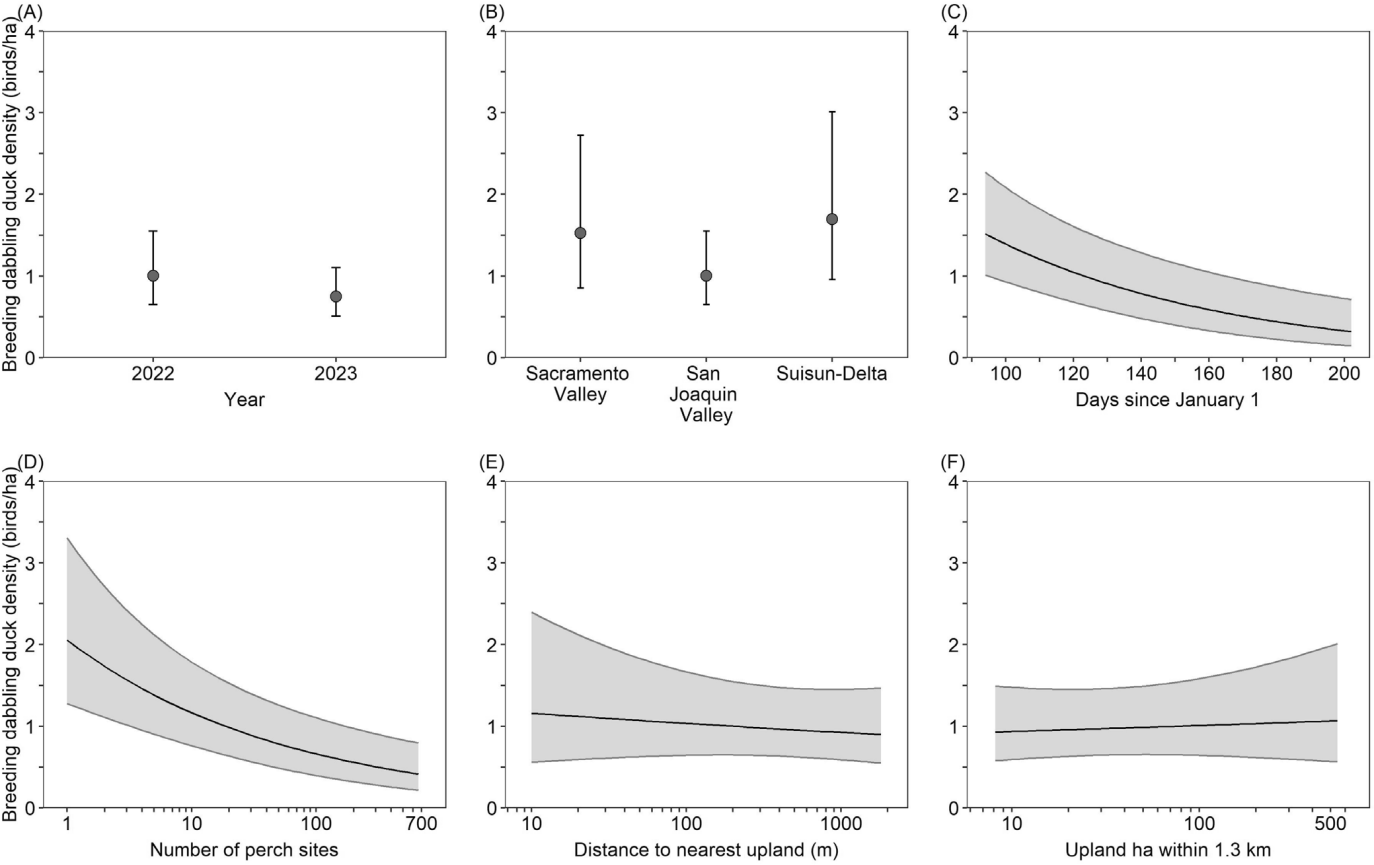
Model‐averaged predictions (±95% confidence intervals) for density of locally breeding ducks on wetlands enrolled in the California Waterfowl Habitat Program during April 3–July 28 as a function of various habitat variables. Predictions (A) and (C–F) were made for the San Joaquin Valley region, by holding all non‐focal continuous variables at their median value (Table 3). Predictions (B–F) were made for the year 2022.

Model‐averaged predictions indicated that between April 3 and July 28, California Waterfowl Habitat Program wetlands in the San Joaquin Valley supported 25% fewer breeding ducks in 2023 than in 2022, and breeding duck density was lower in the San Joaquin Valley (2022: 1.00 ducks/ha, 2023: 0.75 ducks/ha) than in the Suisun–Delta (2022: 1.70 ducks/ha, 2023: 1.27 ducks/ha) and Sacramento Valley (2022: 1.52 ducks/ha, 2023: 1.14 ducks/ha). Breeding duck density decreased 79% from 1.52 ducks/ha on April 4 (day 94) to 0.32 ducks/ha on July 21 (day 202); decreased 80% from 2.06 ducks/ha in wetlands with 1 perch site to 0.41 ducks/ha in wetlands with 710 perch sites; decreased 22% from 1.16 ducks/ha in wetlands located 10 m from the nearest upland habitat > 1 ha in size to 0.90 ducks/ha in wetlands located 1853 m from the nearest upland habitat > 1 ha in size; and increased 15% from 0.93 ducks/ha in wetlands with 8 ha of upland habitat within 1.3 km to 1.07 ducks/ha in wetlands with 582 ha of surrounding upland habitat (Figure 8).

### 3.8 Seasonal Versus Semi‐Permanent Wetlands

**FIGURE 9:**
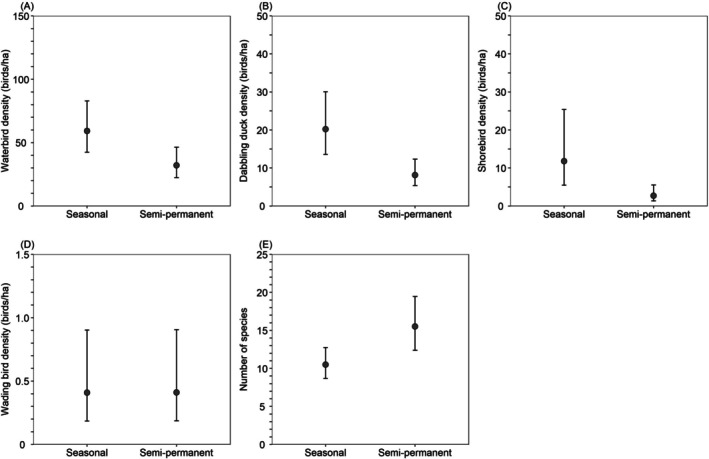
Model‐averaged predictions (±95% confidence intervals) for late winter and early spring (February 3—April 20) density of (A) all waterbirds, (B) dabbling ducks, (C) shorebirds, (D) wading birds, and (E) species richness on wetlands enrolled in the California Waterfowl Habitat Program and managed as either seasonal (flooded in the fall, with standing water maintained throughout the winter until water is drawn down in the spring to support wintering and migrating birds) or semi‐permanent (flooded in the fall or winter with standing water maintained through the summer to support wintering and breeding birds). Predictions were made for the year 2022, for the San Joaquin Valley region; by holding all non‐focal continuous variables at their median value (Table 3).

**FIGURE 10:**
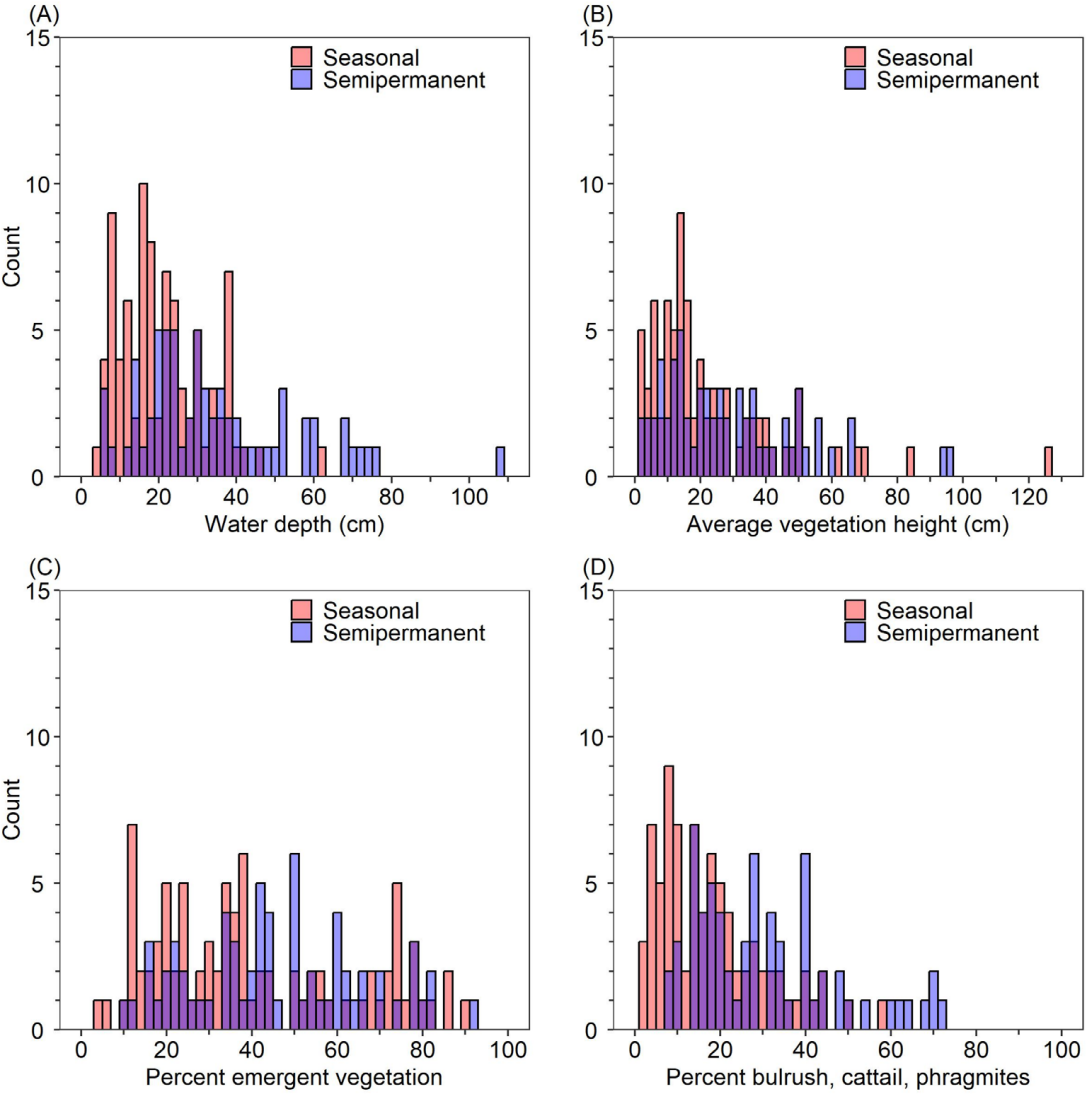
The distributions of (A) average water depth, (B) average vegetation height, (C) percent emergent vegetation cover, and (D) percent tall vegetation cover (stands of bulrush, cattails, and common reed) among seasonal and semi‐permanent wetlands enrolled in the California Waterfowl Habitat Program and surveyed between February 3 and April 20 during 2022 and 2023.

When not considering any wetland‐unit‐specific variables (e.g., water depth, percent emergent cover, and vegetation height were excluded), wetland type influenced the density of all waterbirds, dabbling ducks, shorebirds, and species richness but did not influence wading bird density during late winter and early spring (Table 12). Model‐averaged predictions indicated that California Waterfowl Habitat Program wetlands managed as seasonal wetlands supported 59.3 waterbirds/ha between February 3 and April 20 compared to 32.2 waterbirds/ha for wetlands managed as semi‐permanent wetlands (Figure 9). Wetlands managed as seasonal wetlands also supported more dabbling ducks (20.2 birds/ha) and shorebirds (11.8 birds/ha) than wetlands managed as semi‐permanent wetlands (dabbling ducks: 8.1 birds/ha, shorebirds: 2.7 birds/ha; Figure 9). In contrast, wading bird use of California Waterfowl Habitat Program wetlands was not influenced by whether a wetland unit was managed as a seasonal or semi‐permanent wetland (seasonal wetland: 0.41 birds/ha, semi‐permanent wetland: 0.41; Figure 9). Finally, wetlands managed as semi‐permanent wetlands supported more waterbird species (15.5 species) than wetlands managed as seasonal wetlands (10.5 species; Figure 9). Examination of distributions of habitat metrics found to influence bird density and species richness during winter and early spring (Table 5) showed key differences between seasonal wetlands and semi‐permanent wetlands. Specifically, seasonal wetlands exhibited shallower water (*z* = 11.4, *p* < 0.001), shorter vegetation (*z* = −2.3, *p* = 0.02), and less cover by bulrush, cattail, and common reed stands (*z* = 3.4, *p* < 0.001), but similar emergent vegetation cover (*z* = 0.7, *p* = 0.46) than did semi‐permanent wetlands (Figure 10).

**TABLE 12:** Model selection results for (A) all waterbird, (B) dabbling duck, (C) shorebird, and (D) wading bird density, and (E) species richness within wetlands enrolled in the California Waterfowl Habitat Program in the Sacramento Valley, San Joaquin Valley, and Suisun–Delta regions of California during February 3–April 20 of 2022 (Year 1) and 2023 (Year 2).

Model (base model: study year + ln(flooded area) + wetland unit)	*k*	−2LogL	AIC_c_	ΔAIC_c_	*w*_i_	Evidence ratio
(A) Waterbird density						
+ region + ln(wetland ha within 5 km) + wetland type	8	2189.95	2206.96	0.00	0.06	1.00
+ region + ln(wetland ha within 5 km)	7	2199.29	2214.07	7.12	0.00	35.09
*Base model: study year + ln(flooded area) + wetland unit*	4	2208.36	2216.63	9.68	0.00	126.39
(B) Dabbling duck density						
+ date + ln(distance to nearest developed land) + wetland type	7	1890.50	1905.28	0.00	0.05	1.00
+ date + ln(distance to nearest developed land)	6	1903.79	1916.37	11.09	0.00	255.96
*Base model: study year + ln(flooded area) + wetland unit*	4	1912.69	1920.96	15.69	0.00	2546.95
(C) Shorebird density						
+ region + ln(wetland ha within 5 km) + wetland type + date	9	1439.53	1458.80	0.00	0.26	1.00
+ region + ln(wetland ha within 5 km) + date	8	1453.69	1470.70	11.90	0.00	383.87
*Base model: study year + ln(flooded area) + wetland unit*	4	1472.35	1480.62	21.82	0.00	5.48 × 10^4^
(D) Wading bird density						
+ minutes after sunrise + date + ln(number of perch sites) + ln(wetland ha within 5 km)	8	843.63	860.64	0.00	0.09	1.00
+ minutes after sunrise + date + ln(wetland ha within 5 km) + wetland type	8	848.21	865.22	4.58	0.01	9.85
*Base model: study year + ln(flooded area) + wetland unit*	4	876.51	884.78	24.14	0.00	1.75 × 10^5^
(E) Species richness						
+ date + wetland type	5	999.00	1009.41	0.00	0.05	1.00
+ date	4	1007.53	1015.80	6.39	0.00	24.38
*Base model: study year + ln(flooded area) + wetland unit*	3	1015.60	1021.76	12.35	0.00	481.04

*Note:* “Wetland type” denotes whether the wetland unit was managed as a seasonal wetland or a semi‐permanent wetland. For each metric, all candidate models (*n* = 607) included the variables from the base model (study year + ln(flooded area)) and a random effect (wetland unit). The top model, the top model with wetland type removed, and the null (base) model are presented (for (D) the top model, the top model with wetland type and the null model are presented).

Abbreviations: −2LogL, −2 × log(likelihood); AIC_c_, second‐order Akaike information criterion; evidence ratio, weight of evidence that the model with the lowest AIC_c_ value is better than the current model; *k*, number of parameters in the model; *w*_i_, Akaike model weight or the likelihood of the current model given the data, relative to other models in the candidate set; ΔAIC_c_, difference in AIC_c_ between the top model and the current model.

When considering other wetland‐unit‐specific variables in addition to wetland type, the best‐performing model that included the variable wetland type received little support (ΔAIC ≥ 3.58) in explaining all waterbird density, shorebird density, and wading bird density. However, the top model with wetland type was competitive in explaining dabbling duck density (ΔAIC = 1.26), and wetland type was in the top model explaining species richness (Table 13).

**TABLE 13:** Model selection results for (A) all waterbird, (B) dabbling duck, (C) shorebird, and (D) wading bird density, and (E) species richness within wetlands enrolled in the California Waterfowl Habitat Program in the Sacramento Valley, San Joaquin Valley, and Suisun–Delta regions of California during February 3–April 20 of 2022 (Year 1) and 2023 (Year 2).

Model (base model: study year + ln(flooded area) + wetland unit)	*k*	−2LogL	AIC_c_	ΔAIC_c_	*w*_i_	Evidence ratio
(A) Waterbird density						
+ % tall vegetation + ln(wetland ha within 5 km) + region + shoreline slope	9	2166.11	2185.37	0.00	0.09	1.00
+ % tall vegetation + ln(wetland ha within 5 km) + region + wetland type	9	2169.63	2188.90	3.52	0.02	5.82
*Base model: study year + ln(flooded area) + wetland unit*	4	2208.36	2216.63	31.26	0.00	6.14 × 10^6^
(B) Dabbling duck density						
+ water depth + water depth CV + date + average vegetation height	8	1868.85	1885.85	0.00	0.08	1.00
+ water depth + water depth CV + average vegetation height + wetland type	8	1870.11	1887.11	1.26	0.04	1.88
*Base model: study year + ln(flooded area) + wetland unit*	4	1912.69	1920.96	35.11	0.00	4.21 × 10^7^
(C) Shorebird density						
+ water depth + water depth^2^ + ln(number of perch sites) + average vegetation height	8	1409.37	1426.38	0.00	0.12	1.00
+ water depth + water depth^2^ + ln(number of perch sites) + wetland type	8	1414.93	1431.93	5.56	0.01	16.10
*Base model: study year + ln(flooded area) + wetland unit*	4	1472.35	1480.62	54.25	0.00	6.02 × 10^11^
(D) Wading bird density						
+ % mud + ln(number of perch sites) + minutes after sunrise + ln(orchard ha within 5 km)	8	836.72	853.73	0.00	0.06	1.00
+ % mud + ln(number of perch sites) + ln(distance to nearest upland) + wetland type	8	841.83	858.84	5.11	0.00	12.85
*Base model: study year + ln(flooded area) + wetland unit*	4	876.51	884.78	31.05	0.00	5.53 × 10^6^
(E) Species richness						
+ flooded shape index + wetland type + water depth + water depth^2^	7	966.83	981.61	0.00	0.13	1.00
+ flooded shape index + water depth + water depth^2^ + % tall vegetation	7	973.14	987.92	6.31	0.01	23.45
*Base model: study year + ln(flooded area) + wetland unit*	3	1015.60	1021.76	40.15	0.00	2.5 × 10^8^

*Note:* “Wetland type” denotes whether the wetland unit was managed as a seasonal wetland or a semi‐permanent wetland. For each metric, all candidate models (*n* = 8154 for A–C, E, *n* = 7264 for D) included the variables from the base model (study year + ln(flooded area)) and a random effect (wetland unit). The top model, the top model with the variable wetland type, and the null (base) model are presented (for (E) the top model, the top model without wetland type and the null model are presented).

Abbreviations: −2LogL, −2 × log(likelihood); AIC_c_, second‐order Akaike information criterion; evidence ratio, weight of evidence that the model with the lowest AIC_c_ value is better than the current model; *k*, number of parameters in the model; *w*_i_, Akaike model weight, or the likelihood of the current model given the data, relative to other models in the candidate set; ΔAIC_c_, difference in AIC_c_ between the top model and the current model.

## 4 Discussion

With the loss of natural wetlands, birds and other wildlife have become increasingly reliant on restored and managed wetlands, as well as certain compatible agricultural crops. Public–private partnerships have been a successful tool for promoting wetland conservation and management on private lands and increasing and enhancing wetland habitat availability (Benson et al. 2019; Brasher et al. 2019). Wetlands enrolled in the California Waterfowl Habitat Program are a significant component of the total habitat available to wetland‐dependent birds in the Sacramento Valley, San Joaquin Valley, and the Suisun–Delta regions. Approximately 7962 ha (2022) and 8535 ha (2023) of flooded habitat were enrolled in the California Waterbird Habitat Program (2022: 6480 ha managed as seasonal wetlands, 1482 ha managed as semi‐permanent wetlands; 2023: 7145 ha managed as seasonal wetlands, 1390 ha managed as semi‐permanent wetlands). This represents roughly 8%–9% of the average area of all wetlands (not including flooded agriculture) and 15% of managed wetlands (duck clubs and wildlife refuges) in the Central Valley during February–April between 2005 and 2020 (Donnelly et al. 2022). Extrapolating our density estimates over the total ha of habitat in the Sacramento Valley, San Joaquin Valley, and Suisun–Delta regions, we estimated that during late winter and early spring (February 3—April 20) the California Waterfowl Habitat Program supported approximately 486,607 birds per day in 2022 and 287,302 birds per day in 2023.

### 4.1 Annual and Regional Differences in Bird Density

Bird densities in California Waterfowl Habitat Program wetlands during winter and early spring were consistently greater in 2022 than in 2023. Similar between‐year differences were observed for dabbling duck abundance, shorebird abundance, and species richness obtained from concurrent surveys of winter‐flooded rice fields in the Sacramento Valley (Peterson, Ackerman, Schacter, et al. 2024). This large interannual difference was likely attributable to the dramatically different precipitation levels and wetland availability in the Central Valley between the years 2022 and 2023. In 2022, California was in the third year of a drought (2020–2022), with nearly 100% of the state under drought conditions throughout the year (U.S. Drought Monitor (USDM) National Drought Mitigation Center, Drought.gov). Beginning in December of 2022 and extending through early 2023, California experienced historically high levels of rainfall, ending the drought throughout most of the state. In late March of 2023, the amount of open water in the Central Valley was 45% greater than in late March of 2022 (Point Blue Water Tracker, https://data.pointblue.org/apps/autowater/, accessed September 2024). This greater habitat availability in 2023 compared to 2022 may have allowed birds to spread out among wetlands and agricultural lands, thereby reducing densities observed in any given wetland unit enrolled in the California Waterfowl Habitat Program.

Densities of all waterbirds during late winter and early spring were also higher in California Waterfowl Habitat Program wetlands within the San Joaquin Valley than in the Sacramento Valley or Suisun–Delta (Figure 2). However, this result should not be interpreted as the San Joaquin Valley supported more birds overall than the Sacramento Valley or Suisun–Delta. In fact, according to the midwinter waterfowl survey conducted in January of 2023 (Brady and Weaver 2023), total duck, American coot, and Sandhill Crane (*Antigone canadensis*) abundance in the Sacramento Valley was 393% greater than in the San Joaquin Valley and 72% greater than in the Suisun–Delta region. Thus, the regional differences in densities we observed may reflect more limited wetland availability, concentrating birds in California Waterfowl Habitat Program wetlands within the San Joaquin Valley compared to the Sacramento Valley, which also contains up to 140,000 ha of winter‐flooded rice fields, and the Suisun–Delta during late winter and early spring.

Locally breeding duck densities on California Waterfowl Habitat Program wetlands also varied regionally during the breeding season (April 3 and July 28) and were greater in the Suisun–Delta (1.70 ducks/ha) and Sacramento Valley (1.52 ducks/ha) than in the San Joaquin Valley (1.00 ducks/ha). These regional differences are similar to the results of previous research on breeding duck nest densities. Suisun Marsh is an important breeding site for dabbling ducks in California (McLandress et al. 1996; Ackerman et al. 2014) and supports the highest mallard nest densities among these three regions, followed by the Sacramento Valley, with much lower nest densities reported for the San Joaquin Valley (McLandress et al. 1996).

### 4.2 Water Depth

In studies of waterbird use of wetland habitat, water depth often is found to be a major driver of waterbird abundance, species composition, and species diversity, and wetlands with shallow and intermediate water depths typically exhibit higher bird diversity and densities (Colwell and Taft 2000; Bolduc and Afton 2008). Water depth was among the most influential wetland habitat features influencing bird use of California Waterfowl Habitat Program wetlands, particularly for dabbling duck, shorebird, and secretive marsh bird densities, and species richness (Table 5). Dabbling duck and shorebird densities were highest in wetlands where average water depth was < 20 cm, and densities decreased as water depth increased (Figures 3B and 4B). For dabbling ducks, density decreased 50% from a water depth of 7 cm (28.3 ducks/ha) to a water depth of 30 cm (14.1 ducks/ha). Shorebird density decreased more rapidly with increasing water depth, decreasing 50% from a water depth of 7 cm (22.4 shorebirds/ha) to a water depth of 13 cm (10.9 birds/ha). Shorebird and dabbling duck preferences for shallow to intermediate water depths reflect these species' foraging modes (Safran et al. 1997; Elphick and Oring 1998; Isola et al. 2000; Dybala et al. 2017; Osborn et al. 2017). Winter species richness also decreased slightly with increasing water depths from 12.4 species in wetlands that were 7 cm deep to 10.5 species in wetlands that were 60 cm deep.

In contrast, secretive marsh bird density during spring increased with water depth, from 0.17 birds/ha in wetlands that were 6 cm deep to 0.76 birds/ha in wetlands that were 68 cm deep (Figure 7D). Of the seven secretive marsh birds examined in this study, 6 are waders that typically forage in shallow water, although some species, such as the least bittern, can forage in water as much as 60 cm deep by clinging to emergent vegetation (Poole et al. 2020). In contrast, the pied‐billed grebe forages by diving in deep water (Muller and Storer 2020). Thus, deeper wetlands may exhibit greater secretive marsh bird densities because they provide both the deep‐water habitat for species such as pied‐billed grebes as well as shallow‐water foraging habitat along the wetland shoreline that can be used by rails and bitterns.

There was some support for a negative effect of variation in water depth on the density of all waterbirds, and, to a lesser extent, the density of dabbling ducks during the winter and early spring. Although greater variation in water depth would presumably allow a wetland to accommodate more species by providing a wider range of water depths, the most abundant species during our surveys were dabbling ducks, geese, American coots, and shorebirds, which typically utilize shallow to intermediate water depths (Colwell and Taft 2000; Isola et al. 2000).

### 4.3 Emergent Vegetation

Increasing percent emergent vegetation cover had a small negative effect on winter dabbling duck and winter shorebird densities (Figures 3F and 4E). However, the percent cover of tall, dense stands of hardstem bulrush, cattails, or common reed had a large negative effect on all waterbird density, such that wetlands with 54% cover supported less than a quarter of the density of birds than did wetlands with only 2% cover (Figure 2C). Previous studies have found that wetlands with intermediate vegetation cover supported greater waterbird densities and diversity during winter, spring migration, and during the breeding season, including dabbling ducks and shorebirds (Isola et al. 2000; Webb et al. 2010; Ballard et al. 2021; Kačergytė et al. 2021; Masto et al. 2022). Indeed, the hemi‐marsh concept of managing wetlands that are approximately 50% open water and 50% water with emergent vegetation has been a common management practice since the 1970s (Weller and Spatcher 1965; Masto et al. 2022). A hemi‐marsh provides a more diverse habitat structure that can support birds with varying habitat preferences, whereas wetlands with 0% or 100% emergent vegetation cover would lack habitat structure diversity and thus may support fewer birds. We included a quadratic effect of emergent vegetation cover to test if intermediate levels of emergent vegetation cover supported more birds, but this quadratic effect was not supported by the data. We suspect that because wetland units with high percentages of emergent vegetation cover, particularly those with high percentages of tall, dense stands of hardstem bulrush, cattails, or common reed, supported so few birds, this resulted in a strong decreasing linear relationship with emergent vegetation cover that obscured any increase in dabbling duck density at intermediate levels of emergent vegetation cover.

In contrast, species richness and spring secretive marsh bird density increased with the percent cover of tall, dense stands of hardstem bulrush, cattails, or common reed (Figures 6C and 7C). Greater winter species richness with increasing emergent vegetation cover likely reflects a greater opportunity for wetland units to support species that prefer habitat with emergent vegetation cover. Secretive marsh birds are inconspicuous and often difficult to detect, in part because many occur in thick, dense stands of vegetation. Thus, although wetlands with greater cover of tall, dense vegetation supported lower overall waterbird density, they may be more likely to support secretive marsh birds and other species that prefer tall, dense emergent vegetation cover. Average vegetation height had a similar influence as emergent vegetation cover, with wetlands with taller vegetation exhibiting lower dabbling duck and shorebird densities during late winter and early spring.

### 4.4 Number of Perch Sites

The number of perch sites (trees and utility poles) was an important predictor of shorebird density, wading bird density, and breeding duck density on California Waterfowl Habitat Program wetlands (Table 5). We note that 15 was the highest number of utility poles observed within 50 m of any wetland unit; therefore, wetlands with large numbers of perch sites (> 100) contained a large number of trees. For all except wading bird density in late winter and early spring, the effect of the number of perch sites was strongly negative. Shorebird density in late winter and early spring was 68% lower in wetlands with 1000 perch sites compared to wetlands with a single perch site (Figure 4C), a decreasing trend similar to that observed in golf course ponds and impoundments in Florida (White and Main 2005) and may reflect shorebird avoidance of wetlands that can support and conceal aerial predators. Similarly, breeding duck density was 80% lower in wetlands with 709 perch sites compared to wetlands with a single perch site (Figure 8D). Previous work has demonstrated a negative correlation with nearby forested areas and orchards and breeding mallard abundance in the Central Valley (Newbold and Eadie 2004) that may reflect avoidance of wetlands that support and conceal predators that depredate eggs or ducklings (Sedivy 2001). In contrast, wading bird densities in late winter and early spring increased with the number of perch sites and were 538% higher in wetlands with 1000 perch sites than in wetlands with one perch site (Figure 5C). Because many wading birds (e.g., herons, egrets) roost and nest within trees near wetlands, wetlands with a large number of perch sites nearby may be more attractive to wading birds than wetlands with fewer perch sites.

### 4.5 Surrounding Land Cover

The amount of wetland habitat within 5 km of a wetland unit was the only surrounding land cover variable we examined that consistently influenced bird use of California Waterfowl Habitat Program wetlands (Table 5). This variable had a strong negative effect on the density of all waterbirds and a smaller negative effect on shorebird, wading bird, and secretive marsh bird density. The density of all waterbirds during late winter and early spring was 86% lower in wetlands with 7830 ha of surrounding wetland habitat compared to wetlands with only 213 ha of surrounding wetland habitat (Figure 2D). This result likely indicates a dilution effect, whereby California Waterfowl Habitat Program wetlands have lower bird densities when there is more wetland habitat available within a 5 km area for birds to spread out. In contrast, more isolated wetlands may be more densely populated because nearby alternative wetland habitats are scarce. For breeding ducks, there was a negative relationship between density and distance to the nearest upland and a weak positive relationship between density and the amount of upland area within 1.3 km (Table 5). Because duckling survival decreases as the distance that young broods must travel between upland nesting habitat and adjacent wetlands increases (Ball et al. 1975; Peterson, Ackerman, Hartman, et al. 2024), these results suggest that California Waterfowl Habitat Program wetlands that are closer to potential upland nesting habitat may be more effective at supporting breeding ducks.

### 4.6 Seasonal Versus Semi‐Permanent Wetlands

Maintaining wetlands as seasonal or semi‐permanent influenced winter and early spring bird use of enrolled wetlands. We found that wetlands managed as seasonal supported 84% more waterbirds, 149% more dabbling ducks, and 337% more shorebirds but 32% fewer species during the winter and early spring than wetlands managed as semi‐permanent. This suggests there may be a cost to the quality of winter habitat that is associated with keeping semi‐permanent wetlands flooded through the summer. Because seasonal wetlands are drawn down in the spring and remain dry over the summer, there is more opportunity for management actions (e.g., vegetation control/removal, discing, targeted irrigations for specific vegetation types) to take place ahead of the following winter. Seasonal wetland management also promotes greater densities of moist‐soil plants exploited by dabbling ducks in the winter (Naylor 2002). Moreover, keeping semi‐permanent wetlands flooded later into the summer can promote growth of cattail and bulrush and reduce production of food plants for wintering waterfowl (Heitmeyer et al. 1989). Indeed, we observed that seasonal wetlands exhibited attributes preferred by waterbirds more so than did semi‐permanent wetlands. Water depth, average vegetation height, and the percent tall, dense stands of hardstem bulrush, cattails, or common reed all tended to be lower in wetlands that were managed as seasonal compared to semi‐permanent (Figure 10). Furthermore, when wetland type was allowed to compete with wetland attribute variables such as water depth, average vegetation height, and percent emergent vegetation cover, bird density models that included wetland type received little support. This further suggests that differences in bird densities by wetland type were the result of differences in measured habitat attributes between seasonal and semi‐permanent wetlands. In other words, wetland habitat variables better explained bird density than did the more generalized wetland type variable. The exceptions were in our analysis of species richness, and to a lesser extent, dabbling duck density, suggesting that additional differences between seasonal and semi‐permanent wetlands beyond the wetland habitat attributes we measured influenced differences in dabbling duck density and species richness.

Although semi‐permanent wetlands supported fewer birds per ha in the winter and early spring than seasonal wetlands, they are critically important habitats for breeding waterfowl, especially for ducklings (Chouinard and Arnold 2007; Peterson, Ackerman, Hartman, et al. 2024) during the summer when wetland habitat in the Central Valley is scarce (Central Valley Joint Venture 2020). Moreover, semi‐permanent wetlands comprise a relatively small percentage of the wetlands enrolled in the California Waterfowl Habitat Program (19% in 2022, 16% in 2023). Nevertheless, differences in bird densities and diversity observed between seasonal and semi‐permanent wetlands during the winter and early spring are an important consideration when setting habitat objectives.

## 5 Management Implications

Bird use of wetlands enrolled in the California Waterfowl Habitat Program was influenced by several habitat variables that could be incorporated into wetland management plans and wetland enrollment criteria. First, as has been found in numerous studies, water depth was a major driver of dabbling duck and shorebird densities in wetlands during the winter and early spring. Maintaining shallow water depths of 7–20 cm would support more dabbling ducks and shorebirds. However, approximately 58% of wetland units surveyed in late winter and early spring had water depths ≥ 20 cm at the time of the survey, suggesting that increasing the amount of preferred water depths within these wetlands would benefit these guilds. Although maintaining shallower water depths may exclude species that require deeper water, such species comprised a very small percentage of the birds observed (e.g., diving ducks comprised only 1.2% of all waterbirds observed). Overall, wetland design and irrigation strategies that prioritize areas of shallow and medium water depth that support the more abundant dabbling ducks and shorebirds using these wetlands, but that also incorporate deep‐water areas for secretive marsh birds and diving ducks, would support more diverse waterbird communities. Second, wetlands with a large proportion of emergent vegetation cover and/or a large proportion of tall, dense stands of hardstem bulrush, cattails, or common reed supported fewer total waterbirds and slightly fewer dabbling ducks and shorebirds but more species during the winter and early spring and more secretive marsh birds during the spring. Thus, maintaining wetlands with ~50% emergent vegetation cover or lower would support greater densities of two of the most abundant bird guilds (dabbling ducks and shorebirds). Conversely, maintaining some wetlands with a greater (> 50%) percentage of emergent vegetation cover, particularly tall and dense vegetation cover, would support greater winter species richness and higher densities of secretive marsh birds. Third, wetlands with fewer trees and other perch sites supported more shorebirds during winter and early spring and breeding ducks during the breeding season, but fewer wading birds in winter and early spring. Wetland enrollment criteria that emphasize wetlands with few trees may improve the value of enrolled wetlands for shorebirds and breeding ducks, yet criteria that emphasize wetlands with many trees may improve the value to wading bird communities. Moreover, because we found that the amount of wetland habitat within 5 km of the wetland unit negatively influenced local bird densities within the enrolled wetland, selecting wetlands for enrollment in areas with less wetland habitat nearby might support more birds than selecting wetlands in areas with abundant wetland habitat nearby. However, this could lead to crowding of isolated wetlands and negative density‐dependent effects. Alternatively, prioritizing wetlands nearby existing wetland habitat for program enrollment could create more contiguous patches of wetland habitat over larger areas, thereby increasing overall waterbird abundance with lower densities in individual wetlands. Finally, among regions, waterbird density in the winter and early spring was greater in the San Joaquin Valley than in the Sacramento Valley or Suisun–Delta. Because contemporary region‐wide surveys indicate that winter bird abundance and wetland availability typically is lower in the San Joaquin Valley, the greater densities we observed indicate that limited winter wetland availability in the San Joaquin Valley may be concentrating birds on wetlands enrolled in the California Waterfowl Habitat Program. Therefore, enrollment criteria that prioritize wetlands in the San Joaquin Valley may benefit the waterbirds we studied more than prioritizing enrollment in other regions of the Central Valley.

## Author Contributions

**C. Alex Hartman:** conceptualization (equal), data curation (lead), formal analysis (lead), funding acquisition (supporting), investigation (equal), methodology (equal), project administration (equal), resources (supporting), software (equal), supervision (lead), validation (equal), visualization (equal), writing – original draft (lead), writing – review and editing (equal). **Joshua T. Ackerman:** conceptualization (equal), data curation (supporting), formal analysis (supporting), funding acquisition (lead), investigation (equal), methodology (equal), project administration (equal), resources (lead), software (equal), supervision (supporting), validation (equal), visualization (equal), writing – original draft (supporting), writing – review and editing (equal). **Sarah H. Peterson:** formal analysis (supporting), methodology (equal), software (equal), visualization (equal), writing – review and editing (equal). **Brady L. Fettig:** data curation (supporting), investigation (equal), methodology (equal), project administration (equal), supervision (supporting). **Mark P. Herzog:** conceptualization (equal), data curation (supporting), formal analysis (supporting), funding acquisition (supporting), investigation (equal), methodology (equal), software (equal), visualization (equal), writing – review and editing (equal).

## Conflicts of Interest

The authors declare no conflicts of interest.

## Data Availability

Data that support the findings of this study are openly available in ScienceBase at: https://doi.org/10.5066/P13UJQP5.
